# Diversity of microbes colonizing forages of varying lignocellulose properties in the sheep rumen

**DOI:** 10.7717/peerj.10463

**Published:** 2021-01-11

**Authors:** Mohammad Farhad Vahidi, Javad Gharechahi, Mehrdad Behmanesh, Xue-Zhi Ding, Jian-Lin Han, Ghasem Hosseini Salekdeh

**Affiliations:** 1 Department of Genetics, Faculty of Biological Sciences, Tarbiat Modares University, Tehran, Iran; 2 Human Genetics Research Center, Baqiyatallah University of Medical Sciences, Tehran, Iran; 3 Key Laboratory of Yak Breeding Engineering, Lanzhou Institute of Husbandry and Pharmaceutical Sciences, Chinese Academy of Agricultural Sciences, Lanzhou, China; 4 Livestock Genetics Program, International Livestock Research Institute (ILRI), Nairobi, Kenya; 5 CAAS-ILRI Joint Laboratory on Livestock and Forage Genetic Resources, Institute of Animal Science, Chinese Academy of Agricultural Sciences (CAAS), Beijing, China, Institute of Animal Science, Beijing, China; 6 Department of Systems Biology, Agricultural Biotechnology Research Institute of Iran, Agricultural Research, Education, and Extension Organization, Karaj, Iran

**Keywords:** Sheep, Rumen incubation, Biomass degradation, Microbiome, 16S rRNA gene sequencing

## Abstract

**Background:**

The rumen microbiota contributes strongly to the degradation of ingested plant materials. There is limited knowledge about the diversity of taxa involved in the breakdown of lignocellulosic biomasses with varying chemical compositions in the rumen.

**Method:**

We aimed to assess how and to what extent the physicochemical properties of forages influence the colonization and digestion by rumen microbiota. This was achieved by placing nylon bags filled with candidate materials in the rumen of fistulated sheep for a period of up to 96 h, followed by measuring forage’s chemical characteristics and community structure of biofilm-embedded microbiota.

**Results:**

Rumen degradation for all forages appeared to have occurred mainly during the first 24 h of their incubation, which significantly slowed down after 48 h of rumen incubation, depending on their chemical properties. Random Forest analysis predicted the predominant role of *Treponema* and *Butyrivibrio* in shaping microbial diversity attached to the forages during the course of rumen incubation. Exploring community structure and composition of fiber-attached microbiota revealed significant differential colonization rates of forages depending on their contents for NDF and cellulose. The correlation analysis highlighted the significant contribution of *Lachnospiraceae* and *Veillonellaceae* to fiber degradation in the sheep rumen.

**Conclusion:**

Our findings suggested that forage cellulose components are critical in shaping the pattern of microbial colonization and thus their final digestibility in the rumen.

## Introduction

The human population growth necessitates the higher production of livestock products. Ruminant animals are particularly important in this context, as they can utilize plant fiber through microbial fermentation in their rumen to produce both meat and dairy products. The major constraints in feeding ruminants are high feed costs but low quality of the existing feed resources (Binod et al., 2010). Many of these animals have traditionally relied on their feeding on lignocellulose-based agricultural wastes, a consumption pattern that encourages research and development efforts for sustainable agriculture, particularly in less developed countries (Binod et al., 2010). The symbiotic relationship between ruminant animals and microbiota in their gastrointestinal tract, particularly the rumen, provides an important lignocellulose degrading factory that enables the host to harvest energy from recalcitrant lignocellulosic materials, which are mostly indigestible by the host animal (Krause et al., 2003).

The efficient degradation of plant materials necessitates rumen microbiota to colonize feed particles and establish a dense and complex biofilm on their surface. This physical attachment enables microbial hydrolytic enzymes to be in close contact with lignocellulosic substrates and also grants prolonged residence of microbes in the rumen (Christopherson & Suen, 2013). It is currently unknown to what extent the physicochemical properties of feeds influence their colonization by rumen microbiota or whether rumen microbiota can attach to different lignocellulosic substrates regardless of their chemical compositions. The colonization by rumen microbiota of ingested plant material has been the focus of several investigations. For example, Edwards et al. (2007) and Huws et al. (2013) explored the population dynamics of the bovine rumen microbiome in response to the ingestion of perennial ryegrass. Similarly, Piao et al. (2014) documented the colonization of switchgrass fodder, and Liu et al. (2016) studied the colonization of rice straw and alfalfa hay. They showed that the nature of the forage does affect the microbiome’s composition. Wang et al. (2016) investigated the rumen fermentation and microbial community of lactating dairy cows fed with rice straw and corn silage.

The present study sought to understand the interaction between forage types and fiber-degrading microorganisms and also describe the temporal pattern of colonization rates of forages with varying lignocellulose properties in the sheep rumen. Determining the optimal microbiota colonization of fiber plants in rumen can be important in manipulating ruminal bacteria to better utilize their residues as livestock feeds and ultimately to improve productivity and sustainability in animal husbandry.

To this end, we investigated six fiber plants that have shown high potential to be used as lignocellulose-based agricultural wastes for feeding livestock in Iran. The plants of camelthorn (*Alhagi persarum*), common reed (*Phragmites australis*), date palm (*Phoenix dactylifera*), kochia (*Kochia scoparia*), rice straw (*Oryza sativa*), and Salicornia (*Salicornia persica*) are cultivated in arid and semi-arid regions of Iran, due to their halophyte nature and high resistance of these plants to drought stress. Previous reports have shown the high production and good digestibility potentials under harsh environmental conditioin saccons, such as high salinity and aridity, which make them suitable as forage crops for livestock feeding (Fan et al., 2013; Kafi & Jami-al-Ahmadi 2008; Kazemi, Eskandary & Shamsabadi 2015; Pagter et al., 2005). Rice straw (*Oryza sativa*) is a lignocellulosic waste of rice production at harvest, mainly cultivated in the northern regions of Iran with an almost Mediterranean mild humid climate. Although rice straw is consumed by ruminant animals, its value as a feed is limited to its high content of lignin and silica, which restricts the extent to which it can be degraded in the rumen into fermentable carbohydrate (Sarnklong et al., 2010).

The present study aimed to identify fibrolytic microorganisms, potentially important to fiber degradation in the sheep rumen, and to compare their community attached to forages of varying levels of digestibility in the rumen. We therefore aimed to evaluate whether the rumen microbes have any preference for attachment to forages with different cellulose, hemicellulose and/or varying levels of lignification contents. To this end, we explored the dynamic changes in microbial communities attached to the forages under prolonged incubation in the sheep rumen (e.g., up to 96 h with 24 h sampling intervals). The choice of sheep, as the focus of the study, reflects the fact that Iran houses one of the largest sheep flocks in the world and this animal has a major place in producing high-quality protein and economic stability in our country (Vahidi et al., 2016). We hypothesized that local breeds of sheep with low nutritional requirements and good adaptation to the high fibrous diet can be good candidates to identify the exclusive rumen bacterial populations. We therefore based this study on a fat-tailed and heavy local sheep breed called “Shal breed” for in situ incubation experiment.

## Material & methods

### In situ rumen incubation and sample collection of the forages

All experimental procedures relevant to animals were approved by the Ethics Committee for Animal Experiments of the Animal Science Research Institute of Iran (Approval code: IR124-05-05-007-95004-95006). Rumen cannulation was performed according to the guidelines and regulations of American College of Veterinary Surgeons (ACVS) using a two-stage rumen cannulation technique as described previously (Martineau et al., 2015).

Six common lignocellulosic forages including camelthorn (*Alhagi persarum*, AP; both stem and leaves), common reed (*Phragmites australis*, CR; both stem and leaves), date palm (*Phoenix dactylifera*, DP; leaves), kochia (*Kochia scoparia*, KS; both stem and leaves), rice straw (*Oryza sativa*; cultivar Hashemi, RS; both stem and leaves), and salicornia (*Salicornia persica*, SC; both stem and leaves) were selected for in sacco rumen incubation and degradation analysis. Three adult male fistulated Shal (Iranian native) sheep of body weight between 62–68 kg were used for the study. The animals were housed in a barn and fed a diet of 70% wheat straw and 30% barley-based ad libitum twice daily at 08:00 and 16:30. The animals had free access to drinking water. Feeds were introduced into the rumen via the fistula; the material was first baked at 55 °C for 48 h in an air-circulating oven and was then ground finely enough to pass through a 2 mm sieve. An aliquot of the ground material (5 ±  0.05 g) was packaged within a heat-sealed nylon bag (5 × 10 cm; 50 µm pore size). The six feed samples were simultaneously incubated in duplicate per sheep and replicated in the three animals, which were placed within the rumen shortly after a morning meal; two bags per each feed were removed after 24 h, and a further two after each of 48 h, 72 h, and 96 h.

After removing the bags, in order to analyze fiber breakdown, one of the pair of bags was placed immediately in cold distilled water, severely rinsed for about 5 min, and then transferred to the laboratory where they were washed in a fully automatic washing machine with cold water for 15 min. The non-incubated nylon bags (0 h) were washed in the same manner using washing machine. The other bag rinsed in running distilled water, while rubbing gently between thumb and fingers until the water became clear, and finally squeezed by hands with sterile gloves to remove excess water. The bags were then snap-frozen in liquid nitrogen and stored at −80 °C until required for subsequent DNA extraction and the other one was processed for detergent fiber analysis.

### Microbial dissociation and DNA purification

Microbial cells adhering to the digested material were recovered by suspending the digested feed particulates with a 1:2 (w/v) ratio in dissociation buffer containing 0.1% (v/v) Tween 80, 1% (v/v) methanol and 1% (v/v) tertiary butanol (pH 2), following Pope et al. (2011). The samples were vigorously vortexed every 1–3 min and this step was repeated at least five times. After that, the plant material was sedimented by imposing three rounds of low speed centrifugation, with the supernatant being transferred to a new tube after each spin. Finally, the microbial content was recovered by a rapid centrifugation (12,000 g for 5 min). Microbial DNA was extracted using the QIAamp^®^ DNA Stool Mini Kit (Qiagen, Hilden, Germany). The concentration of the DNA preparations was determined using a NanoDrop spectrophotometer (Thermo Scientific, Wilmington, DE, USA) and its integrity was checked by inspection of an electrophoretically separated (1% agarose gel) aliquot.

### In sacco feed digestion analysis

The neutral detergent fiber (NDF), acid detergent fiber (ADF), and acid detergent lignin (ADL) contents of the digested feed samples were determined following Van Soest, Robertson & Lewis (1991). The measured loss in dry matter (DM), NDF, ADF, and ADL were used as indices of the forage’s degradation. The cellulose and hemicellulose contents of forages were calculated, by subtracting ADL from ADF and ADF from NDF, respectively. In sacco disappearances of the measured contents over sampling periods were calculated as the difference between the amount of those components in the substrates before and after incubation.

### 16S rRNA library preparation and sequencing

A two stage PCR amplification was employed to both enrich for 16S rRNA sequence and add Illumina sequencing adaptors and dual index bar-codes to the sequencing template. Initially, the universal primer pair Bact-0341F (5′-CCTACGGGNGGCWGCAG) / Bact-0785R (5′-GACTACHVGGGTATCTAATCC) was used to amplify a 460 bp fragment corresponding to V3 and V4 regions of the bacterial 16S rRNA sequence (Klindworth et al., 2013). Each 50 µL PCR contained 50 ng DNA (2 µL), 25 µL 2x PCR Master Mix (Thermo Scientific), 21 µL nuclease-free water, 1 µL forward primer (50 pM) and 1 µL reverse primer (50 pM). The PCR condition consisted of an initial denaturation at 94 ° C for 4 min followed by 25 cycles of 94 °C for 30 s, 55 °C for 30 s, and 72 °C for 30 s, and a final extension at 72 °C for 5 min. PCR products were purified and 2 µL of each reaction was used as a template for the second round of PCR during which the Illumina adaptors and barcode sequences were incorporated to the 5′-end of the amplified products. The second PCR was also performed in triplicate under the same running condition except the number of cycles were limited to 15. The amplicons were recovered from a 2% agarose gel and purified using the QIAquick Gel Extraction Kit (Qiagen), quantified fluorometrically, pooled in equimolar quantities, and each sheep individually paired-end sequenced (PE300) using the Illumina MiSeq System at Macrogen Inc. (Seoul, South Korea).

### Sequence analysis

Trimmomatic v0.36 software was used to edit the raw paired-end sequence data, removing adaptor sequences and bases associated with a Phred score <3 (Bolger, Lohse & Usadel, 2014). A four base sliding window was used to scan the reads in order to delete when the average quality per base fell below 15. Paired-end reads were merged using FLASH software (Magoč & Salzberg, 2011) and reads which could not be assembled were discarded. Raw fastq files were de-multiplexed using the split_libraries_fastq.py script of the QIIME pipeline (version 1.9.1) (Caporaso et al., 2010b). Chimeric sequences were removed using the VSEARCH with the most recent version of RDP database (release 11) as a template (Rognes et al., 2016). Sequences were then clustered into operational taxonomic units (OTUs) using UCLUST software (Edgar, 2010), applying a 97% similarity cut-off. The most abundant sequences belonging to each OTU cluster were considered as representative and were aligned against the Greengenes core set (DeSantis et al., 2006) using the PyNAST aligner (Caporaso et al., 2010a). The Ribosomal Database Project naïve Bayesian classifier was used to obtain taxonomic information, applying a minimum support threshold of 80% (DeSantis et al., 2006; Wang et al., 2007).

The OTU table was filtered for low abundant OTUs using filter_otus_from_otu_table.py script with –min_count_fraction option was set to 0.00001 (discarding OTUs represented by <0.001% of the sequences). The OTU table was rarefied to the sequencing depth 3460 reads which corresponded to the number of reads in the sample with minimum number of reads as the sub-sampling depth, then rarefaction curves of the clustered OTUs were drawn using the QIIME pipeline (Caporaso et al., 2010b). Rarefaction curves through alpha diversity indexes were also plotted using high sequencing depth (50000). Alpha diversity indices including Shannon, Simpson, Good’s_coverage, and Chao1 were calculated using core_diversity_analyses.py script in the QIIME pipeline. Beta diversity indices including weighted and unweighted Unifrac phylogenetic distance matrices were constructed with rarefied OTU table as input and visualized through principal coordinate analysis (PCoA) plots made by R/ggplot2 (Wickham, 2016). In the present study, due to uneven sequencing depths across samples, we needed to take low rarefaction value to keep more samples and avoid losing any treatment. The extent to which that could affect the diversity results was assessed through rerunning the alpha and beta diversity analysis using higher sequencing depth (50000).

### Statistical analysis

Statistically significant differences in physicochemical data including DM, NDF, ADF, ADL, cellulose, and hemicellulose contents were analyzed by one-way ANOVA and the general linear model (GLM) procedure of the SAS software v9.3 (SAS Institute Inc., Cary, NC, USA). The main and interaction effects of forage and incubation time were measured based on chemical compositions of six experimental forages. The orthogonal contrast was performed to assess the distributional trend of ruminal microbiome (genus level) adhered to the six lignocellulosic forages during their rumen incubation. *In situ* degradability kinetics for the chemical composition of the experimental forages was evaluated by the exponential model (Orskov & McDonald, 1979).

Differences in taxa abundances between forages and sampling intervals were estimated using analysis of composition of microbes (ANCOM) based on relative abundances of OTUs summarized at various taxonomic levels (Mandal et al., 2015). Means were compared by Duncan post-hoc test in PAST v4.1 given Bonferroni *P*-value cutoff <0.05 (Hammer, Harper & Ryan, 2001). Error correction was made based on the number of groupwise comparisons performed at each taxonomic level. Permutational multivariate analysis of variance (PERMANOVA) was performed using the adonis function of R-package vegan v2.5-5, to test for significant differences between community compositions of forage attached microbial communities. In addition, permutation multivariate analysis of group dispersions (PERMDISP), using the betadisper function of vegan, was used to test for the homogeneity of dispersions (variances). The Spearman correlation analysis was performed using the corr.test function of the psych package v1.8.12 and visualized using R/ggcorrplot package. The *p*-values were corrected using Bonferroni method based on the total number of correlations calculated for each variable separately. For all tests, *p*-values less than 0.05 were considered statistically significant.

### Sequence deposition

All raw amplicon sequences were submitted to NCBI short read archive under BioProjectID: PRJNA642227.

## Results

### Physicochemical characteristics of the incubated feeds

The selected forages were analyzed with respect to their contents for NDF, ADF, ADL, cellulose, and hemicellulose before and after rumen incubation (Table 1, Table S1). They showed a significantly high diversity (*p* < 0.05) concerning their NDF contents within which CR showed the highest (4.39 ± 0.02) but AP and RS showed the lowest (3.93 ± 0.04 and 3.89 ± 0.02, respectively) mean NDF contents (Table 1). The ADF contents of forages varied with AP (3.07 ± 0.02) had the highest and RS (2.33 ± 0.02) but SC (2.37 ± 0.03) had the lowest amounts. With respect to the ADL contents, the differences between forages were also statistically significant (*p* < 0.05). AP (1.25 ± 0.02) showed the highest but RS (0.20 ± 0.006) the lowest amounts of initial ADL while the difference between CR and KS was not significant in this respect. The contents of forages for cellulose and hemicellulose also varied, in which CR and RS showed the highest cellulose while SC and CR showed the highest hemicellulose contents, whereas AP displayed the lowest amounts of both cellulose and hemicellulose among other forages.

**Table 1:** Digestion of six forages after 0, 24, 48, 72, and 96 h incubation in sheep rumen.

Time	Feeds	DM	NDF	ADF	ADL	Cellulose	Hemicellulose
Before rumen incubation	AP	5.04 ± 0.005^a^	3.93 ± 0.04^e^	3.07 ± 0.02^a^	1.25 ± 0.02^a^	1.82 ± 0.04^d^	0.86 ± 0.01^e^
	CR	5.04 ± 0.005^a^	4.39 ± 0.02^a^	2.76 ± 0.02^b^	0.38 ± 0.03^c^	2.38 ± 0.008^a^	1.63 ± 0.04^b^
	DP	5.03 ± 0.01^a^	4.18 ± 0.04^c^	2.68 ± 0.04^c^	0.60 ± 0.02^b^	2.08 ± 0.02^bc^	1.51 ± 0.07^d^
	KS	5.03 ± 0.005^a^	4.07 ± 0.05^d^	2.45 ± 0.06^d^	0.41 ± 0.005^c^	2.04 ± 0.05^c^	1.62 ± 0.01^bc^
	RS	5.02 ± 0.005^a^	3.89 ± 0.02^e^	2.33 ± 0.02^e^	0.22 ± 0.006^e^	2.13 ± 0.01^b^	1.55 ± 0.004^cd^
	SC	5.04 ± 0.01^a^	4.32 ± 0.03^b^	2.37 ± 0.03^e^	0.33 ± 0.006^d^	2.04 ± 0.03^c^	1.95 ± 0.003^a^
24 h after rumen incubation	AP	3.44 ± 0.12^cd^	2.90 ± 0.08^b^	2.35 ± 0.03^a^	0.94 ± 0.03^a^	1.41 ± 0.04^d^	0.55 ± 0.06^e^
	CR	3.68 ± 0.05^b^	3.14 ± 0.03^a^	2.11 ± 0.02^b^	0.34 ± 0.009^c^	1.77 ± 0.01^b^	1.04 ± 0.01^c^
	DP	3.39 ± 0.06^de^	2.96 ± 0.08^b^	2.05 ± 0.06^b^	0.43 ± 0.02^b^	1.61 ± 0.08^c^	0.92 ± 0.02^d^
	KS	3.33 ± 0.10^e^	2.81 ± 0.06^c^	1.85 ± 0.09^c^	0.30 ± 0.02^d^	1.55 ± 0.11^c^	0.96 ± 0.04^cd^
	RS	4.01 ± 0.04^a^	3.24 ± 0.04^a^	2.10 ± 0.03^b^	0.20 ± 0.008^e^	1.89 ± 0.03^a^	1.13 ± 0.02^b^
	SC	3.61 ± 0.04^bc^	3.19 ± 0.06^a^	1.78 ± 0.006^c^	0.29 ± 0.02^d^	1.49 ± 0.007^c^	1.40 ± 0.05^a^
48 h after rumen incubation	AP	3.02 ± 0.21^b^	2.62 ± 0.17^c^	2.20 ± 0.14^a^	0.90 ± 0.09^a^	1.30 ± 0.06^c^	0.42 ± 0.02^d^
	CR	3.58 ± 0.25^a^	3.09 ± 0.25^a^	2.05 ± 0.24^ab^	0.35 ± 0.02^bc^	1.70 ± 0.23^a^	1.04 ± 0.02^b^
	DP	2.95 ± 0.15^b^	2.66 ± 0.08^c^	1.85 ± 0.03^bc^	0.37 ± 0.05^b^	1.48 ± 0.03^b^	0.81 ± 0.06^c^
	KS	3.05 ± 0.09^b^	2.73 ± 0.07^bc^	1.91 ± 0.02^bc^	0.28 ± 0.02^cd^	1.63 ± 0.02^ab^	0.83 ± 0.05^c^
	RS	3.70 ± 0.16^a^	2.98 ± 0.11^ab^	1.93 ± 0.06^abc^	0.19 ± 0.01^d^	1.74 ± 0.05^a^	1.05 ± 0.05^b^
	SC	3.38 ± 0.15^a^	3.06 ± 0.11^a^	1.77 ± 0.03^c^	0.29 ± 0.03^bcd^	1.47 ± 0.03^b^	1.34 ± 0.07^a^
72 h after rumen incubation	AP	2.87 ± 0.13^bcd^	2.66 ± 0.15^ab^	2.14 ± 0.12^a^	0.87 ± 0.07^a^	1.27 ± 0.05^c^	0.52 ± 0.04^e^
	CR	3.40 ± 0.03^a^	2.94 ± 0.04^a^	2.01 ± 0.04^b^	0.37 ± 0.006^b^	1.65 ± 0.04^ab^	0.93 ± 0.001^c^
	DP	2.84 ± 0.14^d^	2.56 ± 0.10^b^	1.76 ± 0.05^c^	0.31 ± 0.02^bc^	1.44 ± 0.04^bc^	0.80 ± 0.06^d^
	KS	2.98 ± 0.14^cd^	2.70 ± 0.13^ab^	1.79 ± 0.09^bc^	0.27 ± 0.02^c^	1.50 ± 0.07^abc^	0.91 ± 0.04^c^
	RS	3.57 ± 0.37^a^	2.89 ± 0.34^ab^	1.84 ± 0.25^bc^	0.18 ± 0.003^d^	1.66 ± 0.24^a^	1.05 ± 0.09^b^
	SC	3.33 ± 0.22^abc^	3.02 ± 0.20^a^	1.68 ± 0.12^c^	0.31 ± 0.008^bc^	1.37 ± 0.12^c^	1.29 ± 0.08^a^
96 h after rumen incubation	AP	2.85 ± 0.15^c^	2.50 ± 0.15^a^	2.14 ± 0.11^a^	0.86 ± 0.04^a^	1.27 ± 0.07^a^	0.37 ± 0.04^d^
	CR	3.20 ± 0.09^b^	2.81 ± 0.11^a^	1.85 ± 0.09^ab^	0.36 ± 0.01^b^	1.50 ± 0.08^a^	0.92 ± 0.02^b^
	DP	2.80 ± 0.12^c^	2.49 ± 0.15^a^	1.69 ± 0.11^bc^	0.30 ± 0.01^c^	1.39 ± 0.09^a^	0.80 ± 0.04^bc^
	KS	2.75 ± 0.06^c^	2.47 ± 0.06^a^	1.73 ± 0.04^bc^	0.26 ± 0.02^d^	1.46 ± 0.02^a^	0.74 ± 0.02^c^
	RS	3.26 ± 0.55^a^	2.64 ± 0.48^a^	1.70 ± 0.31^c^	0.18 ± 0.03^e^	1.52 ± 0.28^a^	0.94 ± 0.18^b^
	SC	3.03 ± 0.48^bc^	2.76 ± 0.44^a^	1.55 ± 0.26^bc^	0.28 ± 0.01^cd^	1.26 ± 0.25^a^	1.22 ± 0.18^a^

**Notes.**

Statistically significant differences were determined using one-way ANOVA. Means were compared using Duncan post-hoc test. Different means were denoted using letters at each time point at *p* < 0.05.

AP camelthorn CR common reed DP date palm KS kochia RS rice straw SC salicornia DM dry matter NDF neutral detergent fiber ADF acid detergent fiber ADL acid detergent lignin.

### In sacco rumen biomass degradation

The six incubated forages were monitored for changes in the contents of DM, NDF, ADF, ADL, cellulose, and hemicellulose during their rumen incubation (Table S2 and Fig. S1). DM degradation was the fastest in KS but slowest in RS (33.8% vs 20.17%, *p* < 0.05) during the first 24 h of rumen incubation. The amount of DM degradation after 48 h of rumen incubation was significantly less than the first 24 h; RS (26.3%) had the lowest degradation but DP (41.3%) had the highest degradation in the second 24 h. With respect to NDF fermentation, RS showed the lowest (16.73%) while KS the highest amount of NDF loss (31.00%) after 24 h of rumen incubation. 48 h after rumen incubation, the highest rate of NDF degradation occurred in DP while the lowest amount was observed in RS. This pattern continued until the end of 96 h of incubation in the rumen. In the first 24 h, besides RS, in which the lowest rate (9.85%) of ADF degradation occurred, all other forages showed a constant rate of degradation (average of 21.2%) and their difference was not significant. 48 h later, a significant difference was observed between DP and RS. After that, up to 96 h, the degradation rate was slight and the differences between the forages were not significant. Regarding ADL degradation, forages were divided into two groups containing AP, DP and KS (average of 25.6%) compared with SC, CR, and RS (average of 7.11%), which had the highest and lowest amounts of average for ADL degradation, respectively. These homogenous subsets showed steadily pattern of degradation over ruminal incubation. In general, by extending the rumen incubation beyond 24 h, the degradation of NDF, ADF, and ADL was significantly declined (Table S2 and Fig. S1). It was apparent that the initial differences in fiber-related physicochemical properties significantly affected the rumen digestion of the six forages. Moreover, forage and incubation time effects significantly affected all fiber-related parameters, however, an interaction effect between forage and incubation time influenced the rumen degradability of ADL and hemicellulose (Table S3).

### Degradation kinetics of chemical compositions

In situ DM, NDF, and ADF degradation kinetic parameters of forages, calculated by an exponential model, are presented in Table S4. There was large variability in soluble fraction (a), slow degraded fraction (b), and degradation rate (C). DM degradability of the forages revealed a low range of the effective degradability (ED) over all experimental forages, on an average of 29.7%, which was the highest in KS (37.50%) following DP (32.70%) but the lowest amount of 16% for RS (*p* < 0.05). The potential degradability also showed a similar tendency with effective degradability, which was over 60% for KS but under 32% for RS. This pattern was in agreement with DM disappearance illustrated in Fig. S1. In general, there was a high range of soluble fraction for NDF (17.51 ± 8.3) and ADF (14.7 ± 7.1) degradation data. Effective degradation (ED2) of NDF ranged from 19.90% to 32.30%, and that of ADF ranged from 15.30% to 28.10%, however, there was no true difference between the forages for ADF and NDF degradability.

### 16S rRNA sequencing data analysis

MiSeq-based sequencing of 16S rRNA gene amplicons resulted in 5,306,118 sequence pairs (average 73,696 pairs of sequences/sample). After quality trimming, sequences were merged using FLASH which resulted in the assembly of 3,967,162 sequences (ranged from 2,729 to 1,34,454 on average of 55,099 sequences/sample) with a mean length 456 bp. A further quality filtering during demultiplexing step discarded 715,635 low quality and short sequences. A total of 839,990 sequences were identified as chimera and discarded. The remaining 2,295,869 non-chimeric sequences were clustered at the 97% sequence identity. After filtering for low abundant features 2,861 clean OTUs representing 2,163,318 sequences were remained for downstream diversity analyses. To test whether our sequencing effort provided sufficient coverage to recover the species diversity of fiber-attached microbiota a rarefaction analysis was performed. The rarefaction curves for most samples approached to plateau but did not reach to the saturation, indicating that our sequencing effort was not enough to fully explore the diversity of forage attached microbiota (Figs. S2 and S3).

### Microbial diversity analysis

**Figure 1:**
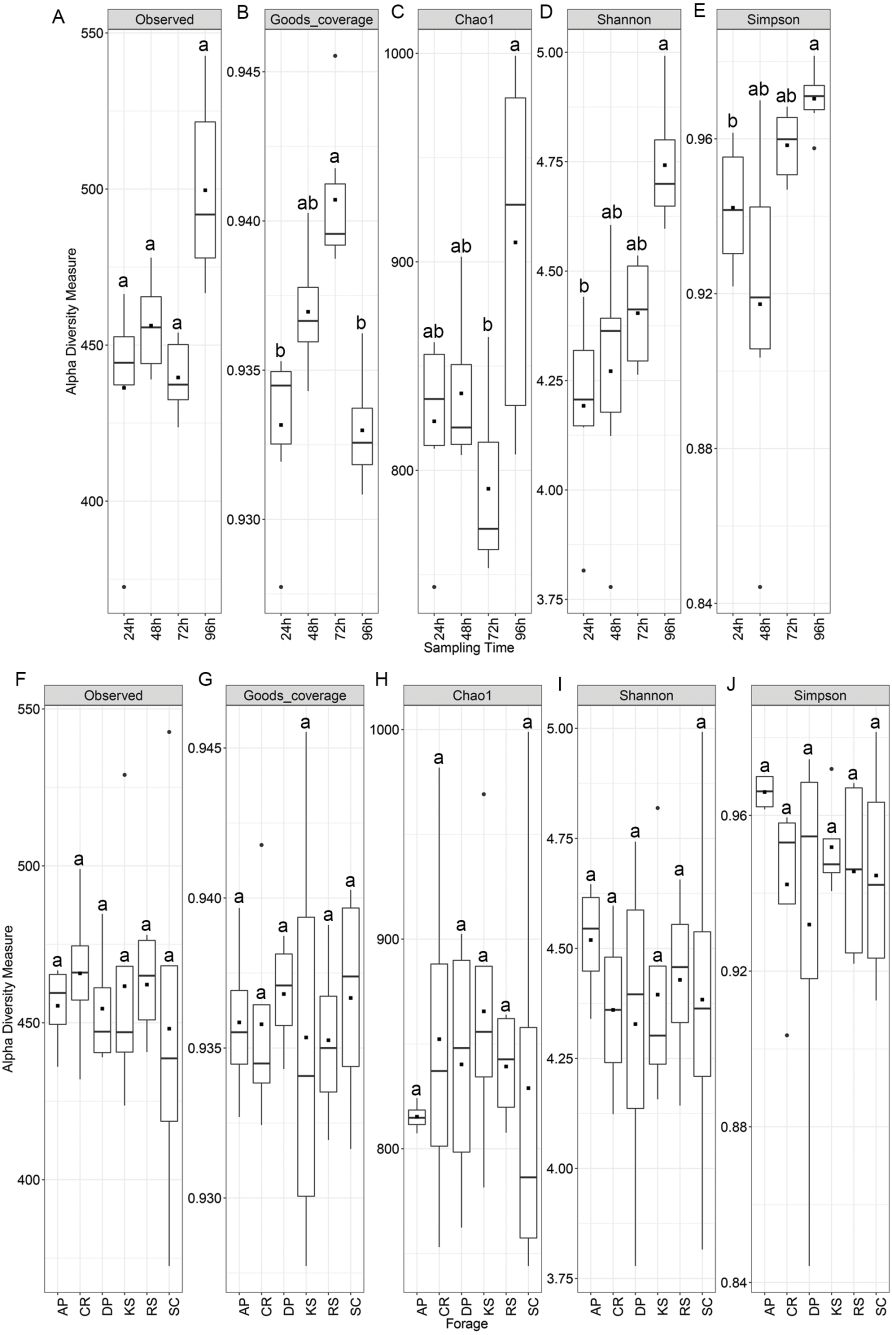
Alpha diversity indices of rumen microbiota attached to the six forages of different lignocellulosic compositions at 24, 48, 72 and 96 h of their rumen incubation. Alpha diversity indices were measured based on OTUs present at an even sequencing depth of 3460 reads (corresponding to the sequencing depth of the sample with the lowest number of reads) in all samples, (A) grouped according to sampling intervals and (B) forages. Statistically significant differences were determined using one-way ANOVA and means were compared by Duncan post-hoc test. Boxplots labeled with different lowercase letters show statistically significant differences. Center line represents median value. AP, camelthorn; CR, common reed; DP, date palm; KS, kochia; RS, rice straw; and SC, salicornia.

Alpha diversity analysis showed no statistically significant difference among the forages but significant variations were observed among sampling intervals (Fig. 1). Chao1 index differed between 72 and 96 h samples, while both Shannon and Simpson indices were significant between 24 and 96 h time points (*p* < 0.05). Nevertheless, diversity indices were not affected by the incubation length within different forages. All samples showed a high Good’s coverage (>0.92, Fig. 1) at the sampled time points, indicating that our sequencing effort allowed to detect >90% of the microbial diversity associated to the forages.

**Figure 2:**
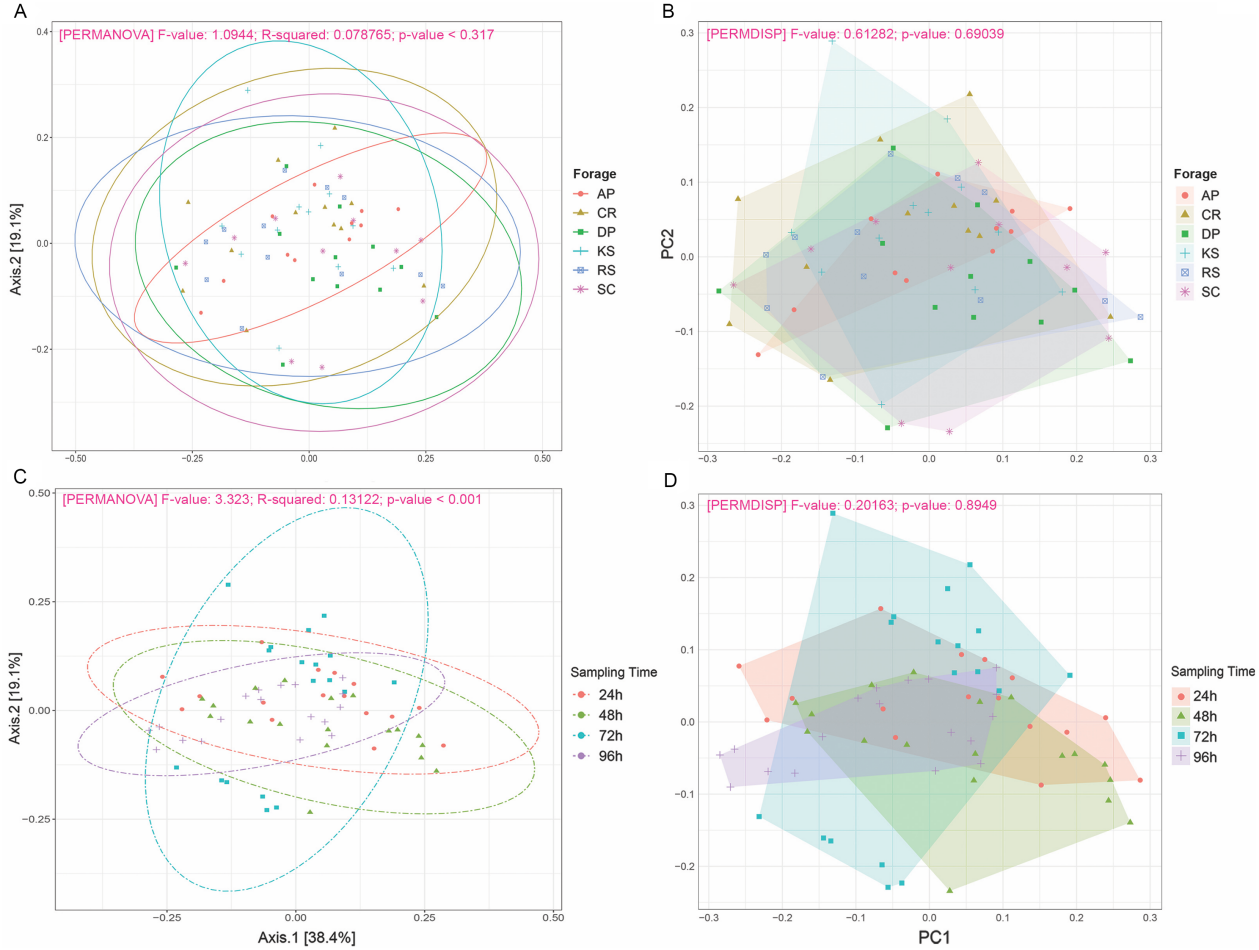
Beta diversity analysis of ruminal microbiome adhered to six different lignocellulosic forages during their rumen incubation. PCoA plots show the distribution of samples based on weighted Unifrac distance matrix in which (A) samples have been grouped according to forages and (B) sampling intervals. Significant differences were tested using PERMANOVA with a *p*-value cutoff 0.01. The percentage of variation explained by each principle coordinate is indicated next to the corresponding axis. The homogeneity of dispersions was also tested for this diversity measure; (C) examining variance differences between forages and (D) sampling intervals. Significant differences were determined using the betadisper function of R package vegan v2.5-5 at 999 permutations. *P*-values less than 0.05 were considered statistically significant. AP, camelthorn; CR, common reed; DP, date palm; KS, kochia; RS, rice straw; and SC, salicornia.

Beta diversity analysis through weighted and unweighted Unifrac divergence indices also revealed no significant difference between forages regardless of their incubation time (Fig. 2). However, when the microbial communities of forage-attached microbes were compared across the incubation length, significant differences were observed in weighted Unifrac (PERMANOVA *P* < 0.001) and unweighted Unifrac (*p* < 0.001) indices (Fig. 2). Testing for homogeneity of group dispersions also revealed no significant difference in dispersions across forages and sampling times (Fig. 2B, PERMDISP *p* > 0.6 and *p* > 0.8, respectively).

In the present study, due to uneven sequencing depths across samples, we needed to take low rarefaction value to keep more samples and avoid losing any treatment. The extent to which that could affect the diversity results was assessed through rerunning the diversity analysis using no-rarefied samples. The outcomes appeared to be identical, so that, significant differences were just observed between the incubation times (Figs. S4 and S5)

### Community composition of forage-attached microbes

**Figure 3:**
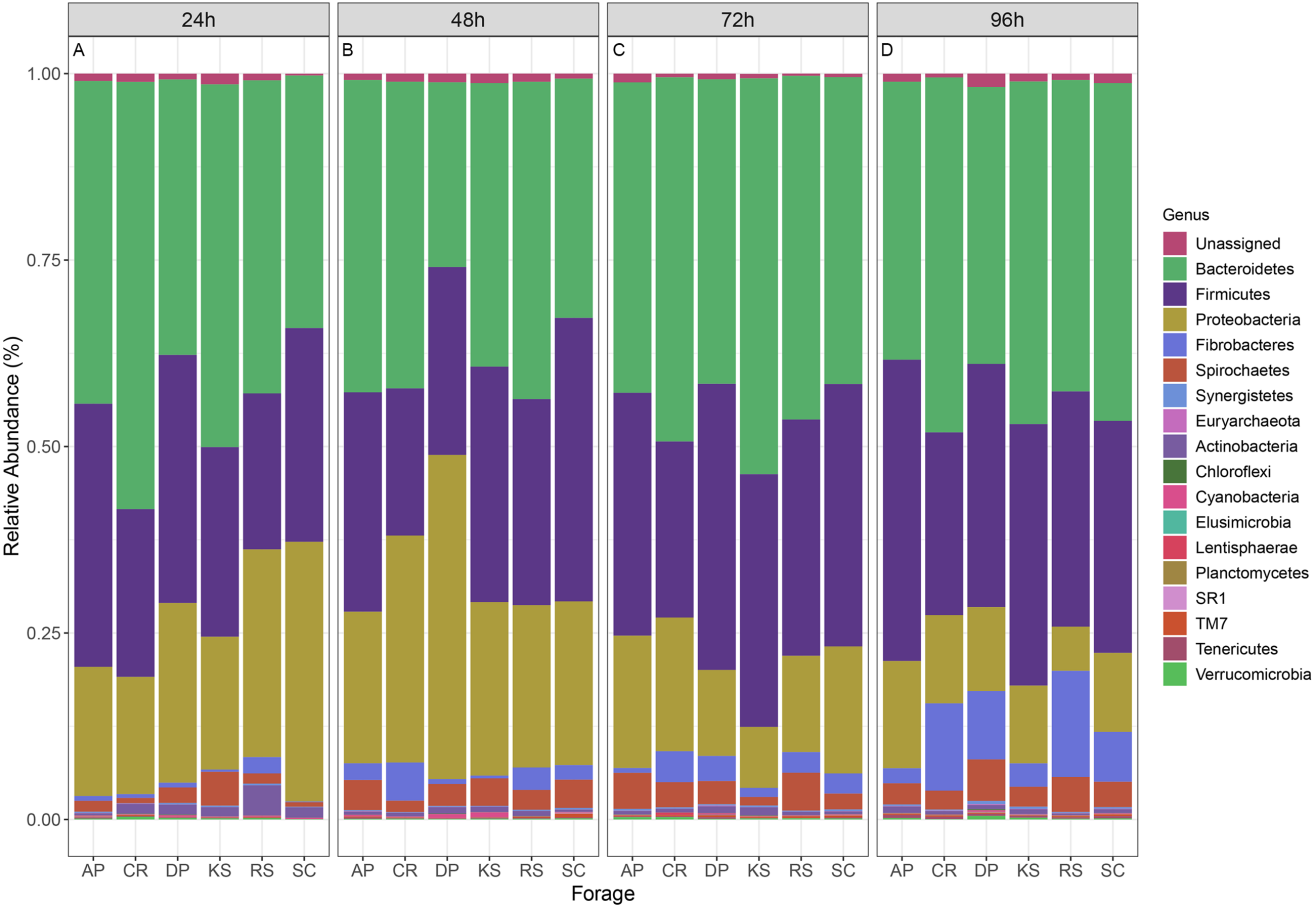
Relative abundance of taxa in different forages. Stacked column bar graph showing the relative abundance of the phyla represented in microbial communities attached to six forages during their rumen incubation. The incubation times over 96 h with 24 h intervals are labeled as (A) 24h, (B) 48h, (C) 72h, and (D) 96h. AP, camelthorn; CR, common reed; DP, date palm; KS, kochia; RS, rice straw; SC, salicornia.

The microbial community associated to forages were assigned to 16 bacterial phyla and one archaeal phylum (Fig. 3). Bacteroidetes (CR and DP) and Firmicutes (CR and AP) showed significant differential abundances between forages, over 96 h ruminal incubation (*p* < 0.05). Bacteroidetes comprised the most abundant phylum accounting for 42.1% of all sequences. Firmicutes were the second most abundant phylum accounting for 30.3% of sequences followed by Proteobacteria (18.5%), Fibrobacteres (3.4%), Spirochaetes (3%), Actinobacteria (0.84%), Synergistetes (0.18%), Verrucomicrobia (0.17%), Tenericutes (0.15%), and TM7 (0.14%). Comparing the prevalence of major bacterial phyla in forage-attached microbes regardless of the incubation time revealed some differences between forages. Bacteroidetes were particularly overrepresented in CR–(48.7%) but were underrepresented in DP-associated microbial community (34.9%). Firmicutes were less represented in CR-attached microbiota (22.6%) while they were equally distributed in other forages (on average 31.9%). Proteobacteria were less represented in the community of KS but overrepresented in that of DP-attached microbes (14.9% and 22.6%, respectively, vs average of 18.3% in other feeds). An interesting finding was the higher prevalence of fiber-degrading bacteria of Fibrobacteres phylum in the microbial community associated with CR (5.4%) and RS (5.5%), two forages with the highest initial cellulose contents, while they accounted for less than 3.3% of reads in other forages.

At the family level, the fiber-attached sheep rumen microbiota was affiliated to 61 bacterial and 2 archaeal families (Fig. S6). Prevotellaceae was the most predominant bacterial family representing about 27.3% of the attached bacterial community followed by Succinivibrionaceae (18%), Lachnospiraceae (13.8%), unclassified Bacteroidales (8.9%), Veillonellaceae (6.2%), Ruminococcaceae (4.1%), Unclassified Clostridiales (4%), and Fibrobacteraceae (3.4%). Bacteroidaceae and Lachnospiraceae were the two families differed between the forages (ANCOM *p* < 0.05; Fig. S7). Bacteroidaceae occurred abundantly in CR and RS-associated microbiota. Members of Lachnospiraceae were less represented in CR- and RS- (*p* < 0.001), while more highly represented in AP-associated microbiota (*p* < 0.02).

**Figure 4:**
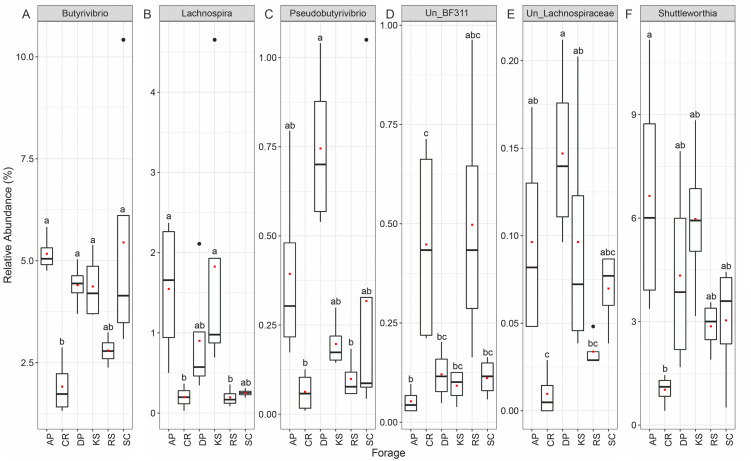
The Relative abundance of taxa (genus level) differentially attached to six different lignocellulosic biomasses following their rumen incubation. Differential abundances were statistically tested using ANCOM with a *p*-value cutoff < 0.05. Means were compared with Dunn’s post-hoc test only accepting Bonferroni corrected *p*-values less than 0.05. Boxplots labeled with different lowercase letters show statistically significant differences. The solid square shows mean and center line represents median value. AP, camelthorn; CR, common reed; DP, date palm; KS, kochia; RS, rice straw; SC, salicornia. Differential taxa are labeled as (A) Butyrivibrio, (B) Lachnospira, C) Pseudobutyrivibrio, (D) Un_BF311, (E) Un_Lachnospiraceae, and (F) Shuttleworthia.

At the genus level, forage-attached microbes were distributed in 106 bacterial genera (Fig. 4), of which, six including *Butyrivibrio*, *Lachnospira*, *Pseudobutyrivibrio*, *BF311*, *unclassified Lachnospiraceae* and *Shuttleworthia* displayed differential abundances among the forages (ANCOM *p* < 0.05, Fig. 4). All members of Lachnospiraceae family showed least significant abundance in CR compared to the other forages (*p* < 0.05). However, unclassified BF311 showed high abundance in CR and RS and they also differed significantly between CR and AP (*p* < 0.05).

**Figure 5:**
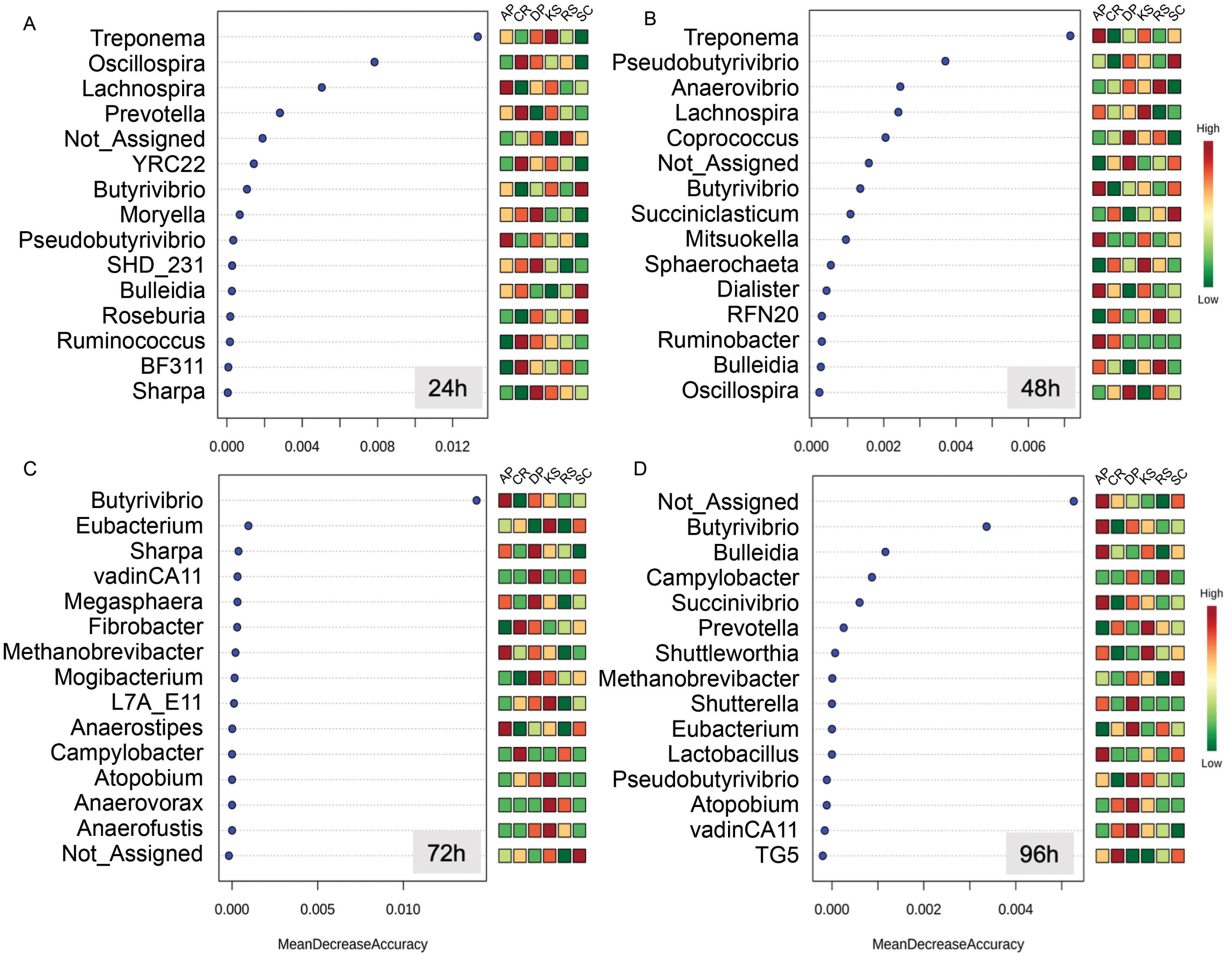
Random Forest (RF) analysis showing the top 15 genera responsible for classification as mean decrease accuracy values. Random Forest classification shows how the relative abundances of different taxa could be used to discriminate the community of microbes attached to different forages during their rumen incubation. Taxa with higher mean decrease in accuracy are stronger predictors of the microbial community attached to feeds. The incubation times over 96 h with 24 h intervals are labeled as (A) 24h, (B) 48h, (C) 72h, and (D) 96h.

The Random Forest machine learning analysis enabled to identify the most predictive taxa discriminating forages from each other during the course of rumen incubation (Fig. 5). During the first 24 h of rumen incubation, *Treponema* was the most predictive taxa for which relative abundances were differed between forages. At this time point, the relative abundances of *Oscillospira* and *Lachnospira* were also helpful for discriminating microbial communities associated to the forages but at a lower degree of accuracy. At the 48 h time point, *Treponema* was also the most predictive taxa followed by *Pseudobutyrivibrio*, *Anaerovibrio*, and *Lachnospira* which their relative abundances could be predictive of microbial communities associated to different forages. At the 72 h time point, *Butyrivibrio* was the only taxa that could be used to separate forages, they showed a significantly low abundance in the forages with the highest initial cellulose contents (e.g., CR and RS). By extending the incubation time to 96 h the prevalence of unassigned sequences was the most predictive one followed by *Butyrivibrio* which was also informative at the previous time point (e.g., 48 h).

### Changes in forage-associated microbes during their rumen incubation

Variations in abundance of genera were also surveyed over the incubation length. The abundance of 14 genera changed with incubation (Fig. S8). Among which, there were several highly abundant rumen bacterial genera which are known to play key roles in plant lignocellulose degradation, including *Fibrobacter, Ruminococcus,* unclassified Lachnospiraceae, unclassified *S24-7,* unclassified Ruminococcaceae, unclassified Veillonellaceae, Lachnospira, Coprococcus, Dialister, Roseburia, Selenomonas, Desulfovibrio, Anaerovibrio, and *RFN20*. The abundance of unclassified Lachnospiraceae gradually increased with incubation length particularly in AP (the forage with the highest initial ADL concentration). The unclassified Ruminicoccaceae were increased with incubation and peaked at 72 h. Members of unclassified Veillonellaceae were more highly represented at 24 h in AP (2.1%) and RS (1.7%) and at 96 h in CR and RS the two forages with the highest initial cellulose concentrations.

**Figure 6:**
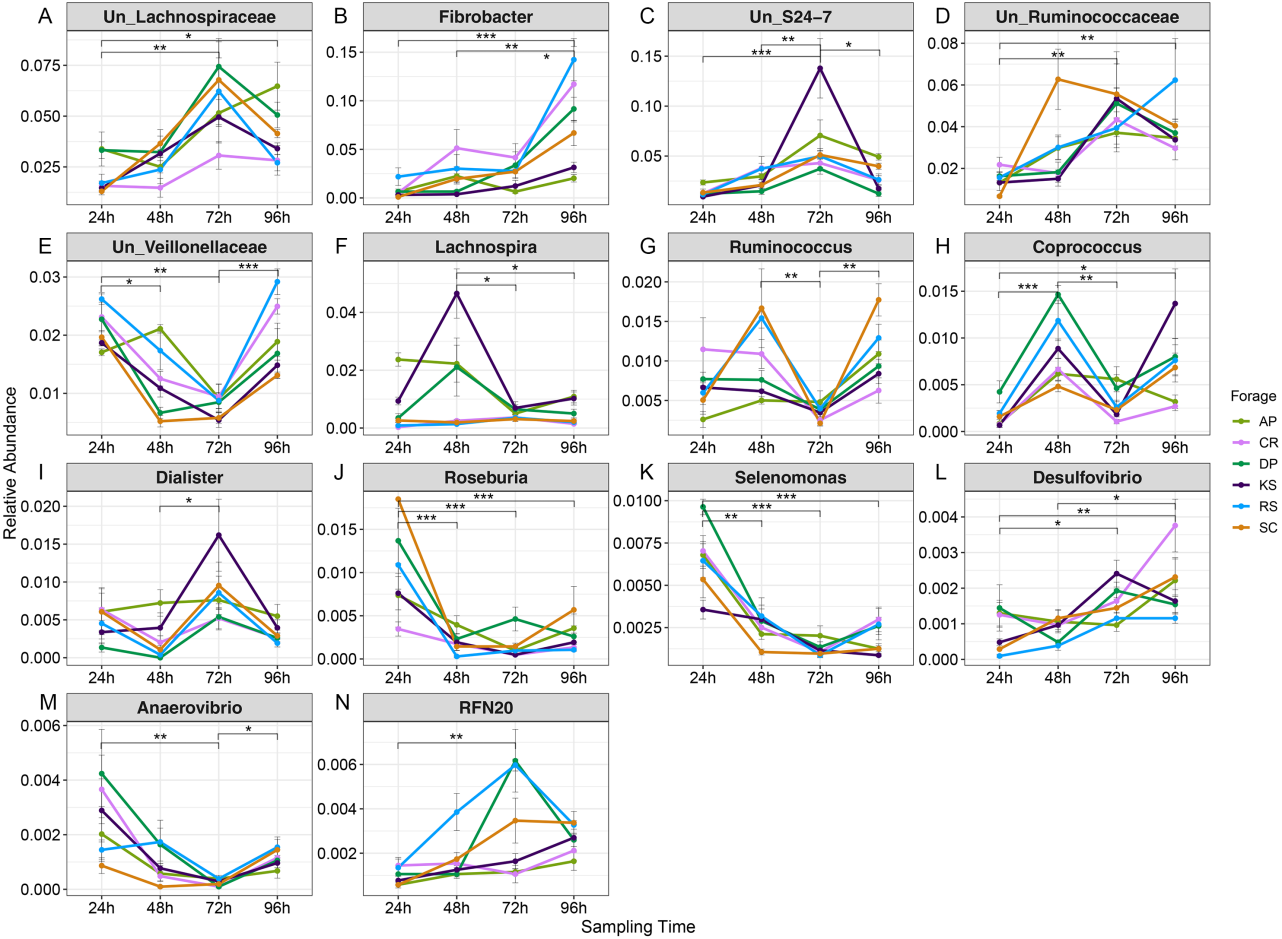
Average relative abundance of genera differentially represented in microbiota attached to six forages during rumen incubation. Line plot to display the average relative abundance of genera differentially represented in microbial communities attached to six forages across rumen incubation (over 96 h with 24 h intervals). Differential abundances were statistically tested using ANCOM with a *p*-value cutoff < 0.05. Means were compared with Dunn’s post-hoc test only accepting Bonferroni corrected *p*-values less than 0.05. Asterisks indicate statistically significant differences between pairs of values (^∗^*P* < 0.05,^∗∗^*P* < 0.01,^∗∗∗^*P* < 0.001). AP, camelthorn, CR, common reed; DP, date palm; KS, kochia; RS, rice straw; SC, salicornia. “Un” refers to unclassified. Differential taxa are labeled as (A) Un_Lachnospiraceae, (B) Fibrobacter, (C) Un_S24-7, (D) Un_Ruminococcaceae, (E) Un_Veillonellaceae, (F) Lachnospira, (G) Ruminococcus, (H) Coprococcus, (I) Dialister, (J) Roseburia, (K) Selenomonas, (L) Desulfovibrio, (M) Anaerovibrio, and (N) RFN20.

Based on orthogonal contrast results (Table S5), Fibrobacters showed a significant linear increase in abundance in RS, KS, DP, and SC during incubation (Fig. 6 and Table S5). The prevalence of *Ruminococcus* and *Coprococcus* cubically changed over nRS rumen incubation, however, *Coprococcus* showed the same trend during DP incubation in rumen (*p* < 0.05). Abundance of *Selenomonas* showed a linearly (AP) and quadratically (CR, DP, and SC) decreasing trends during ruminal incubation (*p* < 0.05). Moreover, *Butyrivibrio* and *Roseburia* indicated linearly decreasing trend in SC (*p* < 0.01) and RS (*p* < 0.05) during ruminal incubation, however, *Roseburia* revealed quadratic variation in abundance during SC incubation (*p* < 0.05) in the rumen. Un_Veillonellaceae and *Treponema* abundances changed quadratically during RS and SC incubation (*p* < 0.01). Un_S24-7 genus linearly increased over SC incubation in rumen, however, its abundance quadratically changed in RS and KS during ruminal incubation.

### The connection between fiber-attached microbiota and lignocellulose degradation

**Figure 7:**
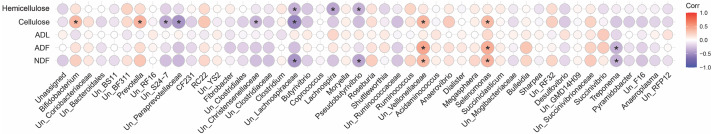
Correlation analysis. Spearman’s correlation analysis between the initial physicochemical properties of the forages and the composition of their attached microbiota at 24, 48, 72 and 96 h of their rumen incubation. Only the genera with more than 0.05 lost dry matter (DM) than the initial amount of DM were considered for correlation analysis. Bonferroni corrected *p*-values (*P* < 0.05) was considered to statistically test the pairwise correlations. Color and size of the circles indicate the magnitude of the correlation, asterisks indicate significance of correlation. Positive correlations are marked in red while negative correlations are marked in blue. NDF, neutral detergent fiber; ADF, acid detergent fiber; ADL, acid detergent lignin.

A spearman correlation analysis was performed to infer the association between digestibility indices and the community composition of fiber-attached microbiota (Fig. 7). This analysis revealed that cellulose content of forages negatively correlated with the abundance of unclassified S24-7 (*r* =  − 0.37, *p* = 0.001), unclassified Paraprevotellaceae (*r* =  − 0.47, *p* = 0.001), unclassified Christensenellaceae (*r* =  − 0.32, *p* = 0.002), and unclassified Lachnospiraceae (*r* =  − 0.53, *p* = 0.002) but positively correlated with the prevalence of *Bifidobacterium* (*r* = 0.37, *p* = 0.001), *Prevotella* (*r* = 0.38, *p* = 0.001), unclassified Veillonellaceae (*r* = 0.38, *p* = 0.001), and *Selenomonas* (*r* = 0.36, *p* = 0.002). The hemicellulose content of the forages was also inversely correlated with the population of unclassified Lachnospiraceae (*r* =  − 0.34, *p* = 0.001), *Lachnospira* (*r* =  − 0.40, *p* = 0.001), and *Pseudobutyrivibrio* (*r* =  − 0.33, *p* = 0.001). Members of unclassified Veillonellaceae (*r* = 0.37, *p* = 0.002; *r* = 0.46, *p* = 0.001) and *Selenomonas* (*r* = 0.39, *p* = 0.001; *r* = 0.42, *p* = 0.001) positively correlated with NDF and ADF concentrations but *Treponema* did inversely (*r* =  − 0.34, *p* = 0.001; *r* = 0.46, *p* = 0.001). Moreover, unclassified Lachnospiraceae (*r* =  − 0.45, *p* = 0.001) and *Pseudobutyrivibrio* (*r* =  − 0.39, *p* = 0.001) correlated negatively with NDF content. The ADL content of forages, however, indicated no statistically significant association.

## Discussion

Here, we investigated biomass degradation and microbial colonization of six lignocellulosic forages during in-situ incubation in the sheep rumen. The selected forages varied in NDF, ADF, ADL, cellulose, and hemicellulose contents, and thus to their rumen digestibility. Forages with the highest initial cellulose concentrations, such as CR (47.2%) and RS (42.43%), experienced the lowest DM loss (26.2% and 20.1% respectively) during the first 24 h of rumen incubation, suggesting that cellulose content is a key factor for biomass degradation in the rumen. The maximum DM degradation for all forages occurred during the first 24 h of rumen incubation, where KS forage lost the highest (33.8%) but RS the lowest (20.1%) of the DM. We also noted an increased DM degradation in the forages with a higher lignin concentration, such as AP (24.8%), as compared to the forages with a lower lignin concentration, such as RS (3.9%). Lignin is resistant to bacterial degradation in the rumen, but the ability of rumen fungi to decompose lignin has already been demonstrated (Raffrenato et al., 2017). Although small amount of lignin can be solubilized, the lignin inhibitory role in the formation of indigestible NDF fraction is thought to be driven by its composition, hydrophobicity, and variations in cross-links between lignin and plant cell wall carbohydrates, which can vary based on the forage types (Raffrenato et al., 2017). Some researchers argued that there are no strong correlations between fibre degradable characteristics and lignin content or lignin to NDF ratio (Coleman, Hart & Sahlu, 2003; Nousiainen et al., 2004; Robinson, Givens & Getachew, 2004). This study showed that the amounts of degraded NDF and ADF had no significant correlation with lignin content.

Gharechahi et al. (2020) studied temporal changes in the microbial communities colonizing the same forages in a native cattle rumen. They reported that RS with the lowest initial NDF had the fastest (66%) while CR with the highest initial NDF had the lowest (42%) DM degradation. However, in the present study, RS showed the least DM degradation. Given the same experimental condition and using the same rice genotype in both studies, the observed differences in degradation pattern is likely fueled by different microbial communities colonizing forages in cattle and sheep rumen and further highlights the key role of rumen microbiota in efficient digestion of plant lignocellulosic substrates.

The disappearance of chemical composition measured on the six experimental forages did not show a clear pattern that explains the rate of DM degradation of the forages being affected by the fiber-related parameters (Table S2). For instance, CR or RS with the highest (4.39%) or lowest (3.89%) initial NDF, respectively, showed the lowest DM degradation during ruminal incubation. Furthermore, in consideration of the lignin inhibitory effect on forage ruminal degradation, AP with the highest initial ADL (1.25%) showed significantly higher DM degradation than RS (0.22%). Overall, these data demonstrate that the detergent system and the chemical composition of the plant are not significant indicators of the rate and extent of digestibility, and that the linkages among fractions are more probably to explain a larger portion of the digestion behavior. Raffrenato et al. (2017) investigated the effect of lignin linkages with other plant cell wall components on in vitro and in vivo NDF digestibility and rate of digestion of grass forages, and demonstrated that, degradability indicators are not significant individually, the chemical structure and relationship between them also play a critical role in the study of performance degradation.

In sacco disappearance and degradation kinetics for the chemical composition of the six experimental forages indicated a partially consistent pattern on DM degradation. Therefore, KS indicated the highest (37.50%) while RS the lowest (16%) effective degradation, but there was no significant difference between the other forages. Disappearance during rumen incubation was more dependent on the potential degradation (A+B) and degradation rate. The degradation kinetics calculated for NDF and ADF parameters demonstrated that despite a significant difference in potential degradation (PD) and degradation rate (c) among forages, the rates of effective degradation were not significant between forages (*p* > 0.05). The R^2^ values for fitted models were 0.78, 0.70, and 0.68 implying reasonable fitting for DM, NDF, and ADF parameters, respectively.

Rarefaction analysis based on indices reflecting species richness and species relative abundances, e.g., Shannon and Simpson (Kim et al., 2017), indicated that the diversity of rumen microbiota attached to the forages had not been sufficiently and evenly sampled. Alpha diversity analysis also showed no biologically significant difference among the forages, which could likely be due to a high microbial heterogeneity among animals included in the study. The observed changes in diversity measures were largely restricted to the initial (24 h) and final hours (96 h) of rumen incubation. Beta diversity analysis also revealed no significant difference between the forages, however, a significant PERMANOVA, but a non-significant PERMDISP, was observed across the length of the rumen incubation, which indicates the presence of a mean effect, instead of the dispersion effect. This could be explained by the fact that although the initial DM degradation resulted in the reduction of digestible components, the accumulation of indigestible residues which had quite similar properties across different forages, favored the attachment of structurally similar communities of rumen microbiota. Huws et al. (2013) also observed similar changes in the microbial communities attached to perennial ryegrass following its incubation in the cow rumen. The extent to which the low rarefaction value could affect the diversity results was assessed through rerunning the diversity analysis using high rarefaction value. The outcomes appeared to be identical, so that, significant differences were just observed between the incubation times.

The microbial communities, attached to forages were affiliated to 16 bacterial phyla. Bacteroidetes and Firmicutes were the two most abundant phyla colonizing forages irrespective of their chemical compositions. This is in accordance with data reported for rice straw, wheat straw, and alfalfa hay which were *in situ* incubated in the cow rumen (Jin et al., 2018; Liu et al., 2016). The third most abundant phylum colonizing forages were Proteobacteria accounting for more than 18% of reads, which is quite high. In the cow rumen, however, Proteobacteria only accounted for 2% of bacteria colonizing rice straw (Liu et al., 2016) and wheat straw (Jin et al., 2018). Particularly, in the cow rumen, Proteobacterial members of the forage epiphytic community were shown to be replaced by members of Bacteroidetes and Firmicutes within 30 min of their rumen entry (Liu et al., 2016). Proteobacteria are known to present in high abundance in the cow rumen fed on high-concentrate diets (Zhu et al., 2017). Members of Firmicutes, Bacteroidetes, Fibrobacteres, Spirochaetes, and Proteobacteria were dominant in all samples, accounting for greater than 96% of the bacterial communities attached to the forages, consistent with the findings for rice straw and alfalfa hay (Liu et al., 2016) and also the forages investigated by Gharechahi et al. (2020). The abundance of these bacterial phyla varied among the forages and across the length of rumen incubation.

It has now been accepted that forage type has a profound impact on the microbial community attached to the forages (Liu et al., 2016). Even different plant parts (e.g., leaves and stems) can attract microbes of varying levels of diversity (Huws et al., 2014). No taxonomic group was found to be specific to a particular forage. This finding suggested that rumen microbiota can attach to forages of varying chemical compositions but with different attachment rates. Similarly, Liu et al. (2016) showed higher bacteria colonization of alfalfa hay than rice straw when the NDF content of the alfalfa hay is lower than that of the rice straw. Thus, the differences between forages in their chemical ingredients determine the extent of their colonization by the rumen microbiota.

Among forage-associated microbes, members of the Firmicutes tend to be less associated with fiber-rich feed CR; whereas, member of Fibrobacteres tend to be strongly associated with CR and RS, as two forages with the highest cellulose contents. The prevalence of members of *F. succinogenes* was linearly increased with incubation of RS, DP, KS, and SC forages in the rumen. Fibrobacteres are known as the main cellulose-degraders of the rumen microbiota and play an important role in the degradation of low-quality fibers (Ransom-Jones et al., 2012; Suen et al., 2011). Members of this phylum were abundantly detected (accounting for more than 5% of sequences) in the microbiota attached to CR and RS. Liu et al. (2016) also reported a positive association between the prevalence of members of this phylum with the content of forages for NDF. In the camel rumen, for example, more than 10% of cellulases and 4.7% of hemicellulases had Fibrobacteres origin even though that their abundance was estimated to be low (<1%) (Gharechahi & Salekdeh, 2018). This finding suggested an unprecedented role for Fibrobacteres in the degradation of cellulose-rich materials in the rumen.

Bacteroidetes are among numerically high abundant members of the rumen microbiota which are best recognized for their saccharolytic activities. The presence of a high number of pectinolytic and cellulolytic enzymes in their genomes clustered with other lignocellulose degrading enzymes into polysaccharide utilization loci (PUL), suggests that they are also actively involved in lignocellulose degradation (Gharechahi et al., 2020; Lapébie et al., 2019). Within this phylum, Bacteroidaceae were significantly overrepresented in the forages with the highest initial cellulose contents (e.g., CR and RS). At the genus-level, however, this differential abundance was only affiliated to the BF311, an uncultured and unknown rumen bacterium. However, correlation analysis showed that the metabolic capability of unclassified BF311 might be similar to the genus *Prevotella*, and the genus unclassified BF311 might participate in VFA metabolism (Bi et al., 2018).

Within Firmicutes phylum, Lachnospiraceae were significantly underrepresented in CR and RS. Members of the Lachnospiraceae family are gram-positive anaerobic rod-shaped bacteria that mainly ferment pectin (Bach et al., 2019; Cotta & Forster, 2006). At the genus-level, this differential abundance was only attributed to *Butyrivibrio*, *Lachnospira*, *Pesudobutyrivibrio*, unclassified Lachnospiraceae, and *Shuttleworthia*. Grilli et al. (2013) reported that rumen bacteria *Pesudobutyrivibrio xylanivorans* and *P. ruminis* exhibit an enzyme system specialized in the degradation of hemicellulose within the goat rumen and hemicellulolytic bacteria group represented a major part of total bacteria in goats fed on a native forage diet. Recently, Huws et al. (2016) reported that DM degradation was speeding up when the population of *Pseudobutyrivibrio*, *Roseburia*, and *Ruminococcus* spp. increased and the population of *Succinivibrio* spp. decreased in perennial ryegrass incubated in the cow rumen. Piao et al. (2014) also observed an increased occurrence of *Pseudobutyrivibrio* and *Ruminococcus* spp. during secondary wave of switchgrass colonization by rumen bacteria of Friesian cow. It is believed that microbial communities attached to forage during the primary phase utilize soluble and easily accessible nutrients while those colonizing forages during the secondary phase are considered to be the true lignocellulose degraders (Huws et al., 2016; Liu et al., 2016). Butyrivibrio spp. are known for their proteolytic, hemicellulolytic, and biohydrogenation activities (Krause et al., 2003). Liu et al. (2016) reported a strong positive correlation between the abundance of Butyrivibrio spp. and the crude protein contents in the rice straw and alfalfa hay, suggesting their preference for adhesion to proteinaceous components of the forages. However, the *Butyrivibrio*/*Pseudobutyrivibrio* group is phylogenetically diverse (Kopečný et al., 2003), which makes it difficult to draw more accurate conclusions about the possible significance of organisms belonging to this group in fiber digestion.

*Shuttleworthia*, a genus belonging to the family Lachnospiraceae, was less represented in the microbial community attached to CR, the forage with the highest initial NDF and cellulose concentrations. This genus has been previously found to be more abundant in ruminal liquids and solids of highly efficient cows (Jewell et al., 2015) and also reported as digesta-adherent rumen bacteria in dairy and beef cattle (Mi et al., 2017; Noel et al., 2017). Carroll (2017) reported the role of *Shuttleworthia* in increasing butyrate fermentation in alfalfa hay compared with grass hay, which was in agreement with reports of enhanced rumen butyrate concentrations and increased *Shuttleworthia* abundance in more efficient cattle (Guan et al., 2008). Members of Lachnospiraceae family were significantly less represented in CR and RS. They are known for their pectin fermentation ability (Cotta & Forster, 2006) and also their polysaccharide degrading function (Deusch et al., 2017) in the rumen. Deusch et al. (2017) indicated an increased abundance of polysaccharide degrading species of the Firmicutes including the families of Lachnospiraceae and Ruminococcaceae in fiber-rich solid phase as confirmed by OTU and protein abundance levels suggesting their significant contribution to lignocellulose degradation in the rumen.

Analysis of the community dynamic of fiber-attached microbiota revealed a decreased abundance of the genera *Roseburia*, *Anaerovibrio*, and *Selenomonas* during the length of rumen incubation independent of fiber type. In contrast, the abundance of Fibrobacters increased as the incubation time extended. But genera, such as unclassified Ruminococcaceae, unclassified Lachnospiraceae, unclassified Veillonellaceae, *Coprococcus* and *Desulfovibrio* did not show a clear pattern. A non-specific colonization behavior indicates that these taxa have the potential to express a broader CAZyme repertoire and adapt to diverse fiber (Moraïs & Mizrahi, 2019). Piao et al. (2014) observed in incubated switchgrass that some genera with less or without fiber-degradation potential including *Anaerostipes*, *Coprococcus*, *Oscillospira*, *Succiniclasticum* and *Desulfovibrio* colonize fiber, have the same timeframe as fiber degraders, suggesting that these taxa benefit from or interact with the fiber degraders and have thus developed and maintained the potential to tightly adhere to the fiber (Moraïs & Mizrahi, 2019). This is in agreement with previous studies demonstrating that around 70% of the bacterial cells in the rumen are attached to plant fiber (Forsberg & Lam, 1977). *Roseburia* and *Coprococcus* may contribute to the production of butyrate, and these two genera seem to be suppressed by a forage-rich diet (Kim et al., 2014). *Anaerovibrio* are known to be associated with lipid degradation (Henderson, 1971) and are abundant in cattle fed corn but are rarely detected in cattle fed with a forage-rich diet.

*Treponema* were underrepresented in the microbiota attached to CR and SC, while they were significantly enriched in the KS-attached microbiota. They also negatively correlated with NDF contents of forages. *Treponema* are known to contribute to pectin degradation in the rumen (Liu et al., 2015). An increase in abundance of members of *Treponema* was also noted in cow rumen microbiota associated with switchgrass after 6 h of rumen incubation (Piao et al., 2014). Early reports on rumen *Treponema* community suggested that they cannot derive on cellulose as a sole carbon source, but they are closely associated with cellulolytic bacteria and utilize carbohydrates released during fiber degradation (Stanton & Canale-Parola, 1980). A recent analysis of the camel rumen metagenome showed that Spirochaetes, which *Treponema* belongs, contributes to more than 7% and 5% of cellulase and hemicellulase gene catalog, respectively, suggesting that they have likely key contribution to lignocellulose degradation in the rumen (Gharechahi & Salekdeh, 2018).

Among forage attached microbes, members of *Prevotella*, *Selenomonas*, and unclassified Veillolenaceae displayed a positive correlation with the residual NDF, ADF, and cellulose contents of forages. *Selenomonas* has been previously detected with high frequency in the community of microbes attached to fiber (Koike et al., 2003). *Selenomonas* cannot degrade cellulose directly, but they can use the cellobiose obtained from the cellulose degradation to produce propionate through the succinate pathway (Wang et al., 2018), to avoid an imbalance of homeostasis in the biochemical pathways in the ruminal environment (Castillo-González, Burrola-Barraza & Domínguez-Viveros, 2014). Members of the Veillonellaceae are capable of utilizing and converting lactate to acetate and propionate (Duncan et al., 2002). Therefore, with the degradation of plant cell-wall (NDF, ADF, and cellulose) by fiber-degrading bacteria, the abundance of Veillonellaceae for utilizing the degradation by-products increases. In a study by Liu et al. (2016), *Prevotella* was found to be positively associated with the content of feeds for crude protein and their abundance was high in the alfalfa hay than rice straw. Species of *Prevotella* are the most abundant taxa in the rumen environment and are known for their starch-degrading and proteolytic activities (Krause et al., 2003). *Prevotella* has been reported to be the most abundant xylanolytic bacteria in the rumen, and thus performing an important role in fiber degradation (Dodd, Mackie & Cann, 2011). A recent analysis of rumen metagenome showed that members of the family Prevotellaceae contribute to more than 45% of CAZymes and thus are likely to play a more significant role in carbohydrate degradation in the rumen (Gharechahi & Salekdeh, 2018). Unclassified S24-7 revealed a negative correlation with the residual content of cellulose. In a recent study, S24-7 bacteria were considered to be a member of the core gut community of sheep (Wang et al., 2017). Population genomics studies have shown that S24-7 family members are encoded for starch-degrading *α*-amylases and thus are capable of starch utilization (Ormerod et al., 2016; Serino et al., 2012). The capacity of the S24-7 family for niche partitioning and/or divergent spatial organization of its members has been shown in a recent study (Ormerod et al., 2016).

## Conclusion

In summary, our results demonstrated that the physicochemical compositions of the forages can potentially drive primary and advance colonization behaviors of microbiota and thus lignocellulose degradation in the sheep rumen. Primary colonization happened during initial hours of forage incubation during which microbiome compete to attach forages for degrading easily digestible components and/or utilize the byproducts. As incubation time extended, advanced colonization behaviors are formed and a relatively uniform microbial community with almost higher relative abundance adhere to the surface of lignocellulosic forages. The cellulose content of the forages was among the factors influencing microbial attachments and lignocellulose degradation in the rumen. However, our data demonstrated that the chemical compositions of the forages were not significant indicators of the rate and extent of digestibility, and that the linkages among fractions should be considered. No taxonomic lineage was found to be specific to a particular forage, suggesting that most rumen microbes have an intrinsic trend for attachment to lignocellulosic substrates.

##  Supplemental Information

10.7717/peerj.10463/supp-1 Supplemental Information 1 Disappearance curves for the six different lignocellulosic forages as a function of incubation time (A-F) AP, camelthorn, CR, common reed; DP, date palm; KS, Kochia, RS, rice straw; SC, Salicornia, NDF; neutral detergent fiber, ADF; acid detergent fiber, and ADL; acid detergent lignin.

10.7717/peerj.10463/supp-2 Supplemental Information 2 Rarefaction curves depicting through alpha diversity indexes using low rarefaction value (A) Rarefaction curves showing the increase in number of observed OTUs; (B) Species richness (number of observed OTUs + number of unobserved OTUs, Chao1); (C) Shannon and (D) Simpson diversity indices on *Y*-axis as a function of the number of reads sampled on *X*-axis.

10.7717/peerj.10463/supp-3 Supplemental Information 3 Rarefaction curves depicting through alpha diversity indexes using high rarefaction value (A) Curves showing the increase in number of observed OTUs; (B) Species richness (number of observed OTUs + number of unobserved OTUs, Chao1); (C) Shannon and (D) Simpson diversity indices on *Y*-axis as a function of the number of reads sampled on *X*-axis.

10.7717/peerj.10463/supp-4 Supplemental Information 4 Alpha diversity indices of rumen microbiota attached to the six forages of different lignocellulosic compositions at 24, 48, 72 and 96 h of their rumen incubation Alpha diversity indices were measured based on OTUs present at an even sequencing depth of 50000 reads in all samples, (A) grouped according to sampling intervals and (B) forages. Statistically significant differences were determined using one-way ANOVA and means were compared by Duncan post-hoc test. Boxplots labeled with different letters show statistically significant differences. Center line represents median value. AP, camelthorn, CR, common reed; DP, date palm; KS, Kochia; RS, rice straw; and SC, Salicornia.

10.7717/peerj.10463/supp-5 Supplemental Information 5 Beta diversity analysis of ruminal microbiome adhered to six different lignocellulosic forages during their rumen incubation PCoA plots show the distribution of samples based on weighted Unifrac distance matrix in which (A) samples have been grouped according to forages and (B) sampling intervals. Significant differences were tested using PERMANOVA with a *p*-value cutoff 0.01. The percentage of variation explained by each principle coordinate is indicated next to the corresponding axis. Significant differences were determined using the betadisper function of R package vegan v2.5-5 at 999 permutations. *P*-values less than 0.05 were considered statistically significant. AP, camelthorn, CR, common reed; DP, date palm; KS, Kochia; RS, rice straw; and SC, Salicornia.

10.7717/peerj.10463/supp-6 Supplemental Information 6 The relative abundance of taxa in rumen microbiota attached to forages Stacked column bar graph showing the relative abundance of taxa (family level) represented in microbial communities attached to six forages during their rumen incubation (over 96 h with 24 h intervals). AP, camelthorn, CR, common reed; DP, date palm; KS, Kochia; RS, rice straw; and SC, Salicornia.

10.7717/peerj.10463/supp-7 Supplemental Information 7 The Relative abundance of taxa (family level) differentially attached to six different lignocellulosic biomasses following their rumen incubation Differential abundances were statistically tested using ANCOM with a *p*-value cutoff < 0.05. Means were compared with Duncan post-hoc test only accepting Bonferroni corrected *p*-values less than 0.05. Boxplots labeled with different letters show statistically significant differences. The solid square shows mean and center line represents median value. AP, camelthorn, CR, common reed; DP, date palm; KS, Kochia; RS, rice straw; and SC, Salicornia.

10.7717/peerj.10463/supp-8 Supplemental Information 8 The Relative abundance of the genera attached to six different lignocellulosic biomasses following their rumen incubation with a sum greater than 0.15 (AA-BE) The solid square shows mean and center line represents median value. AP, camelthorn, CR, common reed; DP, date palm; KS, Kochia; RS, rice straw; and SC, Salicornia. “Un” refers to unclassified.

10.7717/peerj.10463/supp-9 Supplemental Information 9 Chemical composition analysis of six original forages (%DM) NDF; neutral detergent fiber, ADF; acid detergent fiber, ADL; acid detergent lignin

10.7717/peerj.10463/supp-10 Supplemental Information 10 *In sacco* disappearance of chemical composition measured on the six experimental forages DM; dry matter, NDF; neutral detergent fiber, ADF; acid detergent fiber, ADL; acid detergent lignin. Statistically significant differences were determined using one-way ANOVA. Means were compared using Duncan post-hoc test. Different means were denoted using letters at each time point at *p* < 0.05. AP, camelthorn, CR, common reed; DP, date palm; KS, Kochia; RS, rice straw; and SC, Salicornia.

10.7717/peerj.10463/supp-11 Supplemental Information 11 GLM ANOVA results (F and *P* values) for the main and interaction effects of forage and incubation time on measured chemical compositions of six experimental forages DF; degree of freedom, DM; dry matter, NDF; neutral detergent fiber, ADF; acid detergent fiber, ADL; acid detergent lignin.

10.7717/peerj.10463/supp-12 Supplemental Information 12 Degradation kinetics for chemical composition of the six experimental forages evaluated by the exponential model (Orskov & McDonald, 1979) *p* = *a* + *b* (1 –exp^−*ct*^) where, p; is rumen disappearance at time t (h), a; washing losses, soluble or rapidly degradable fraction constant, b; slowly degradable fraction constant, c; degradation rate, t; time of incubation, ED; effective degradability was calculated as a + (b × c)/(c + k) at three ruminal passage rates (*k* = 0.02, 0.04, and 0.06 h^−1^), PD; potential degradability calculated as a+b. Statistically significant differences were determined using one-way ANOVA. Means within row, were compared using Duncan post-hoc test. Different means were denoted using letters for each forage at Bonferroni corrected *p* < 0.05. AP, camelthorn, CR, common reed, DP, date palm; KS, Kochia; RS, rice straw; and SC, Salicornia. DM; dry matter, NDF; neutral detergent fiber, ADF; acid detergent fiber, SEM; standard error of the mean.

10.7717/peerj.10463/supp-13 Supplemental Information 13 Testing for distributional trend of ruminal microbiome (genus level) adhered to the six lignocellulosic forages during their rumen incubation (over 96 h with 24 h intervals) using orthogonal contrast analysis Linear _*i*_ ; linearly increased, Linear _*d*_ ; linearly Kochia; RS, rice straw; and SC, Salicornia.

## Additional Information and Declarations

### Data Availability

The following information was supplied regarding data availability: The data are available at NCBI SRA: PRJNA642227.
