# Supplementary figures and images for: Diversity of microbes colonizing forages of varying lignocellulose properties in the sheep rumen
Source: PeerJ. 2021 Jan 11;9:e10463. doi: 10.7717/peerj.10463 (PMC7808268; doi:10.7717/peerj.10463)

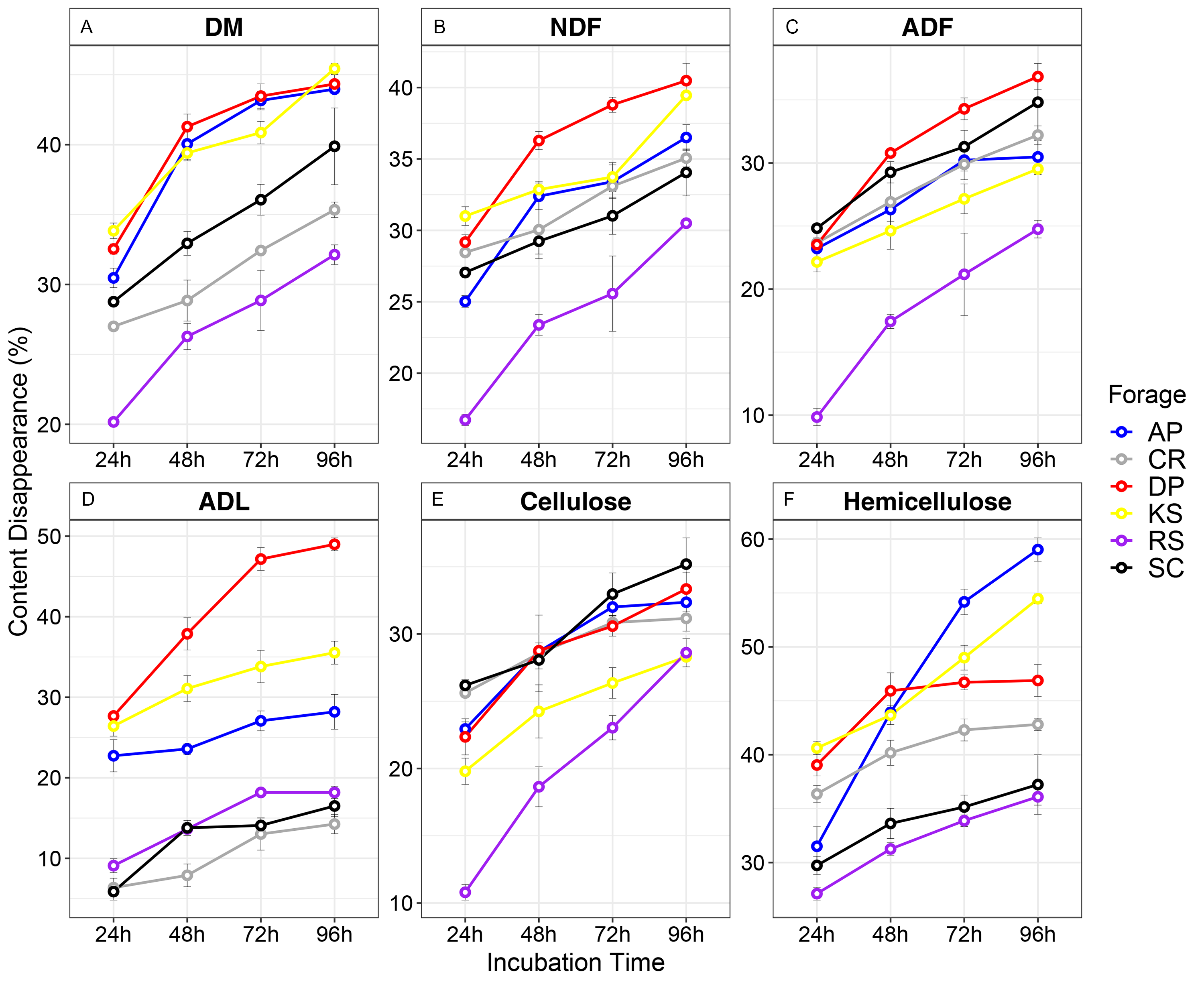

Supplement: Supplemental Information 1 — AP, camelthorn, CR, common reed; DP, date palm; KS, Kochia, RS, rice straw; SC, Salicornia, NDF; neutral detergent fiber, ADF; acid detergent fiber, and ADL; acid detergent lignin. [file peerj-09-10463-s001.png]

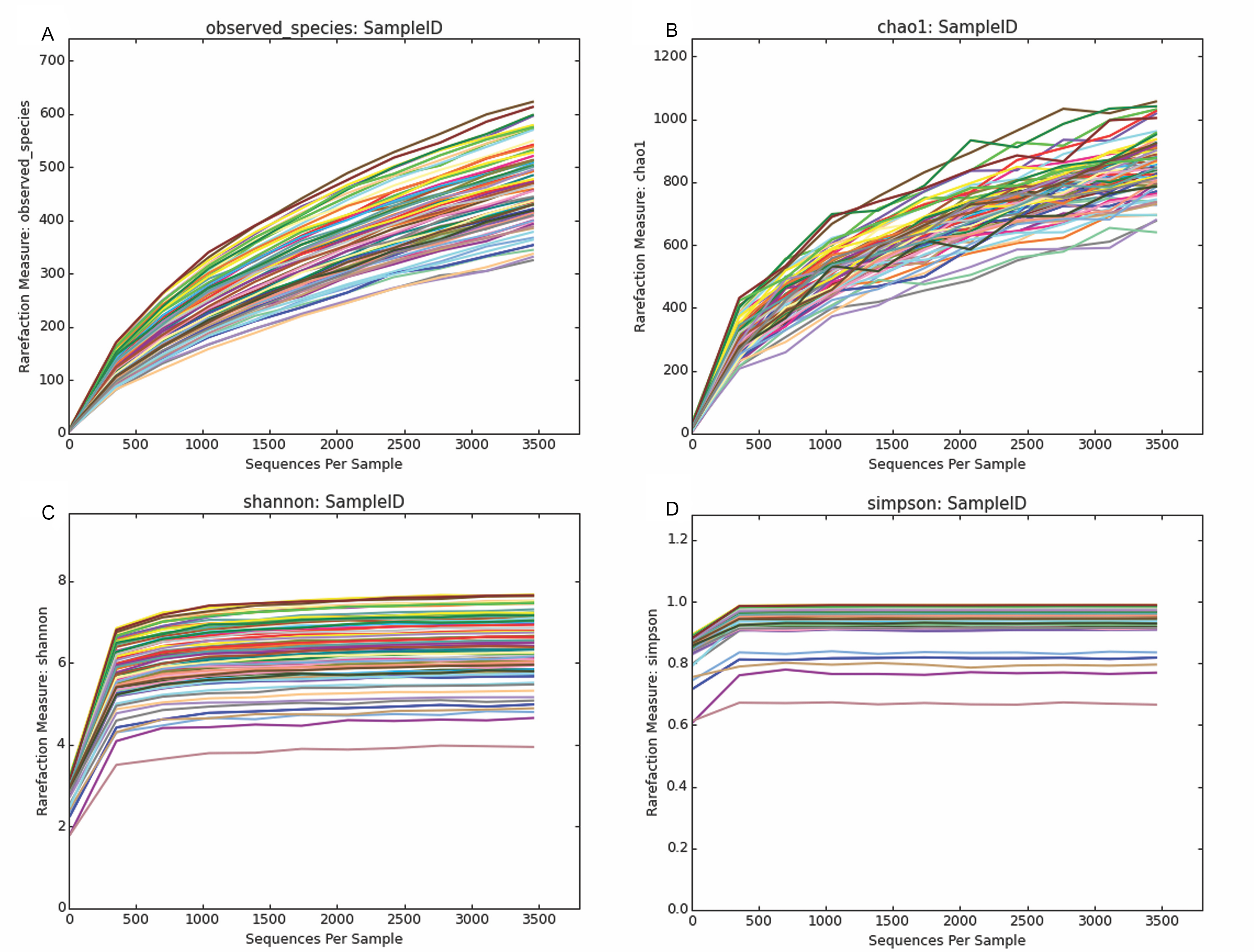

Supplement: Supplemental Information 2 — (A) Rarefaction curves showing the increase in number of observed OTUs; (B) Species richness (number of observed OTUs + number of unobserved OTUs, Chao1); (C) Shannon and (D) Simpson diversity indices on Y-axis as a function of the number of reads sampled on X-axis. [file peerj-09-10463-s002.png]

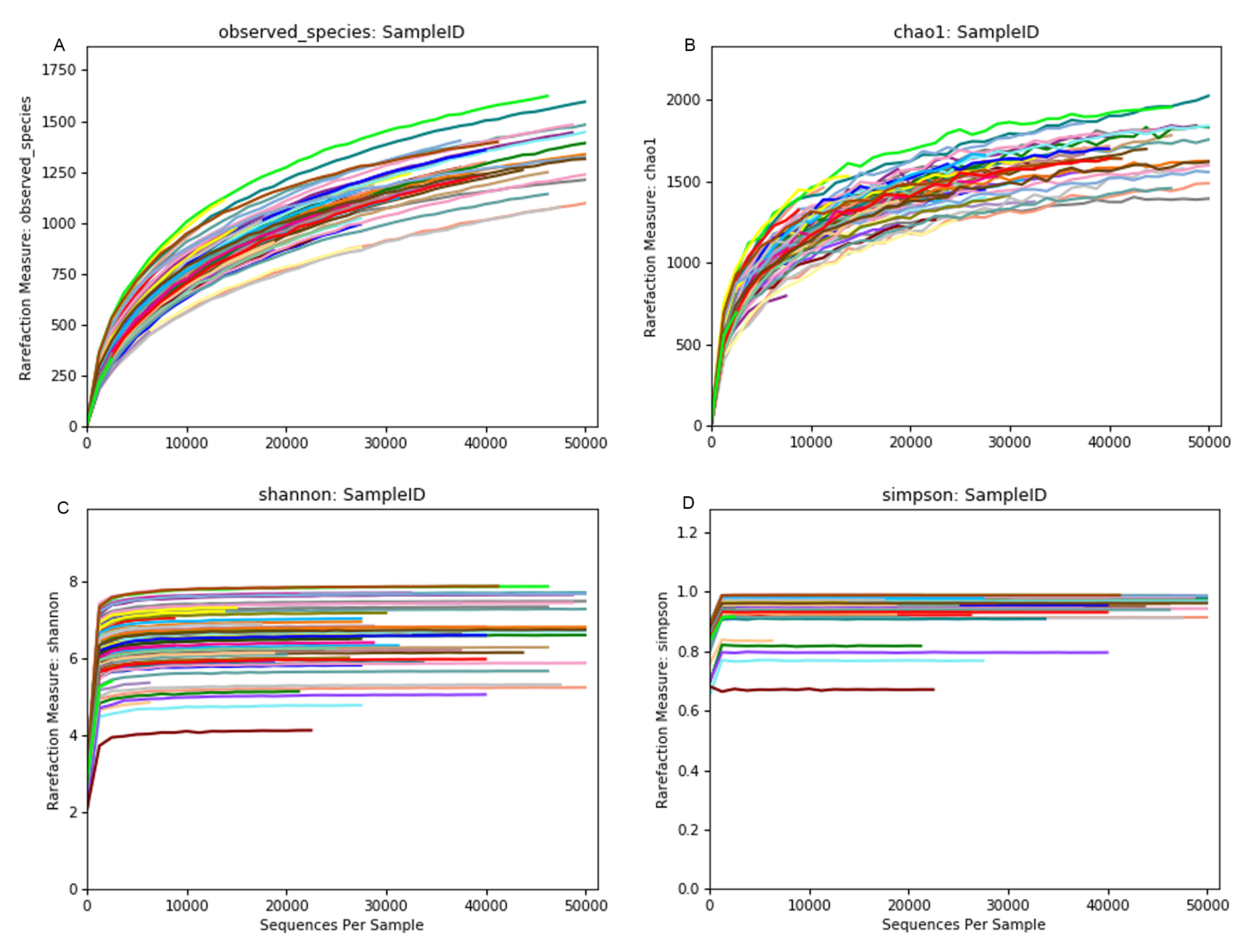

Supplement: Supplemental Information 3 — (A) Curves showing the increase in number of observed OTUs; (B) Species richness (number of observed OTUs + number of unobserved OTUs, Chao1); (C) Shannon and (D) Simpson diversity indices on Y-axis as a function of the number of reads sampled on X-axis. [file peerj-09-10463-s003.png]

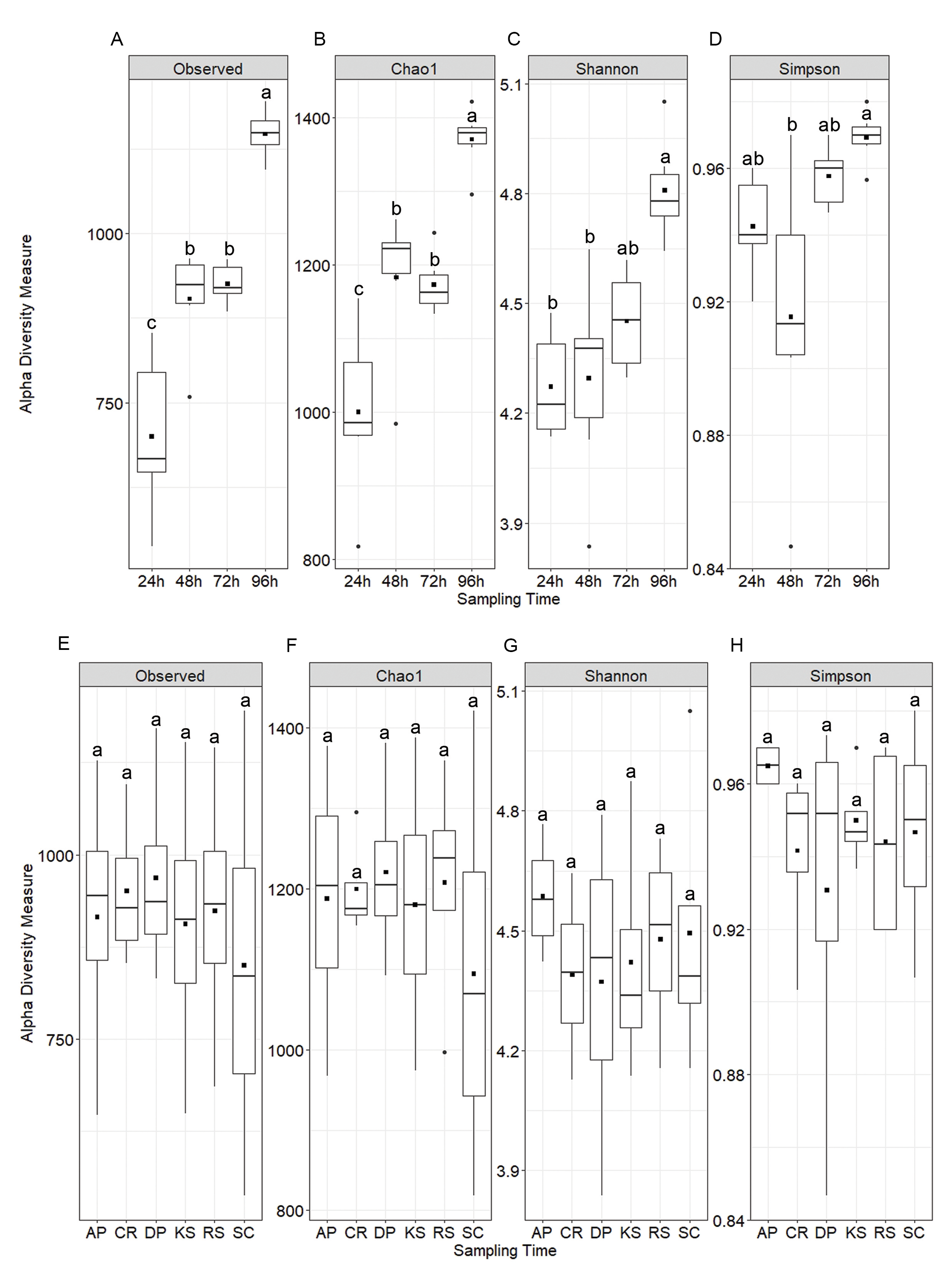

Supplement: Supplemental Information 4 — Alpha diversity indices were measured based on OTUs present at an even sequencing depth of 50000 reads in all samples, (A) grouped according to sampling intervals and (B) forages. Statistically significant differences were determined using one-way ANOVA and means were compared by Duncan post-hoc test. Boxplots labeled with different letters show statistically significant differences. Center line represents median value. AP, camelthorn, CR, common reed; DP, date palm; KS, Kochia; RS, rice straw; and SC, Salicornia. [file peerj-09-10463-s004.png]

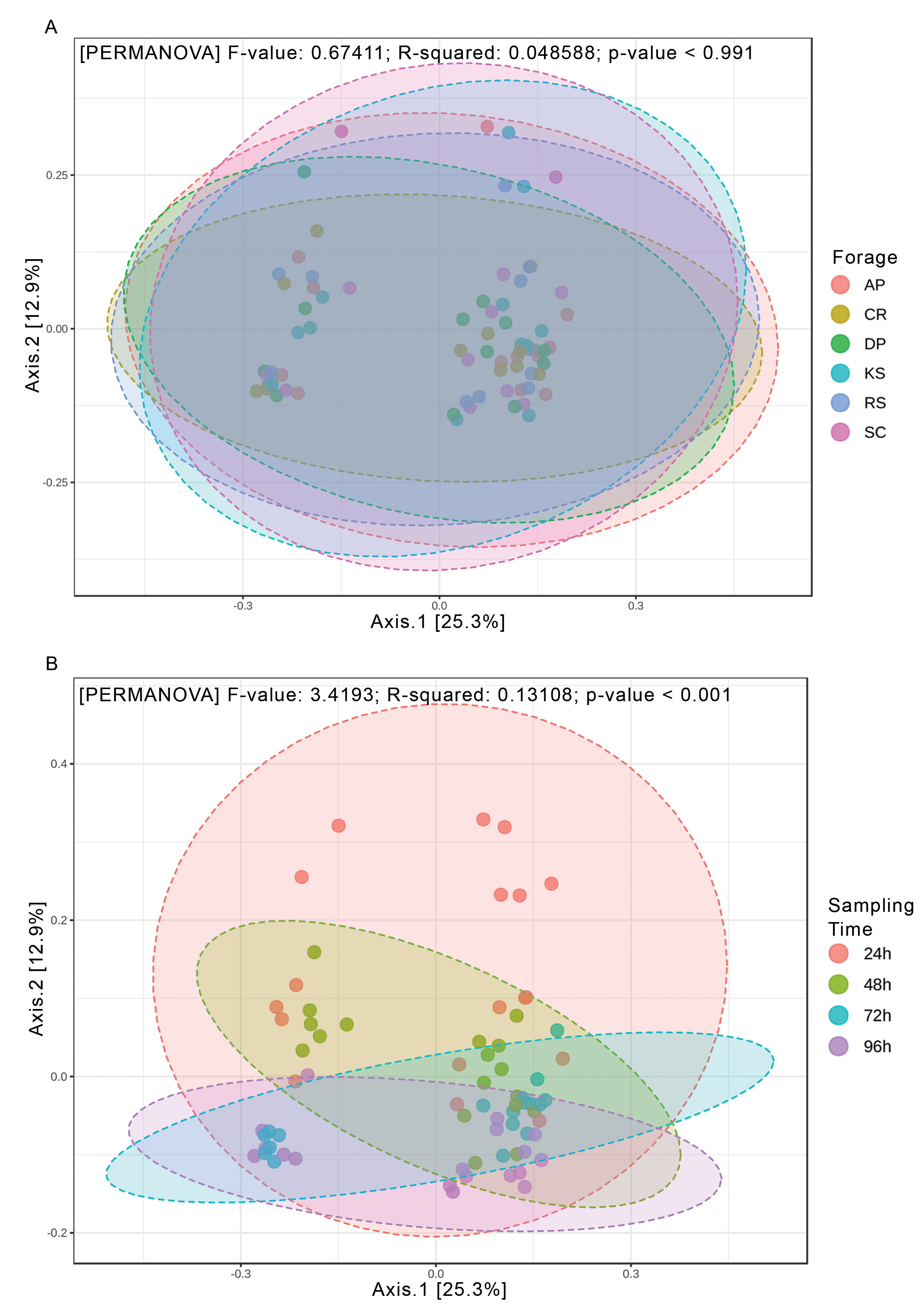

Supplement: Supplemental Information 5 — PCoA plots show the distribution of samples based on weighted Unifrac distance matrix in which (A) samples have been grouped according to forages and (B) sampling intervals. Significant differences were tested using PERMANOVA with a p-value cutoff 0.01. The percentage of variation explained by each principle coordinate is indicated next to the corresponding axis. Significant differences were determined using the betadisper function of R package vegan v2.5-5 at 999 permutations. P-values less than 0.05 were considered statistically significant. AP, camelthorn, CR, common reed; DP, date palm; KS, Kochia; RS, rice straw; and SC, Salicornia. [file peerj-09-10463-s005.png]

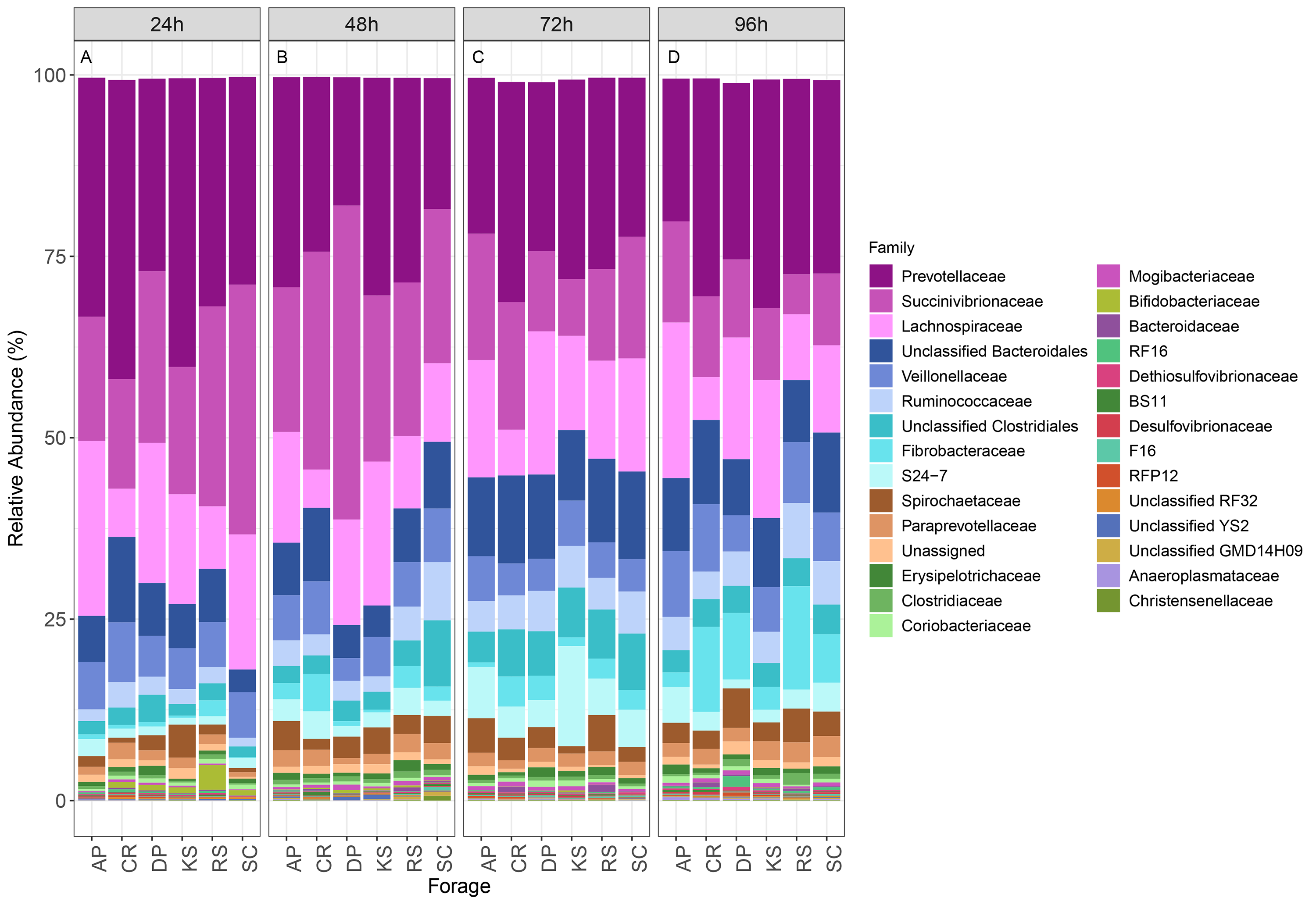

Supplement: Supplemental Information 6 — Stacked column bar graph showing the relative abundance of taxa (family level) represented in microbial communities attached to six forages during their rumen incubation (over 96 h with 24 h intervals). AP, camelthorn, CR, common reed; DP, date palm; KS, Kochia; RS, rice straw; and SC, Salicornia. [file peerj-09-10463-s006.png]

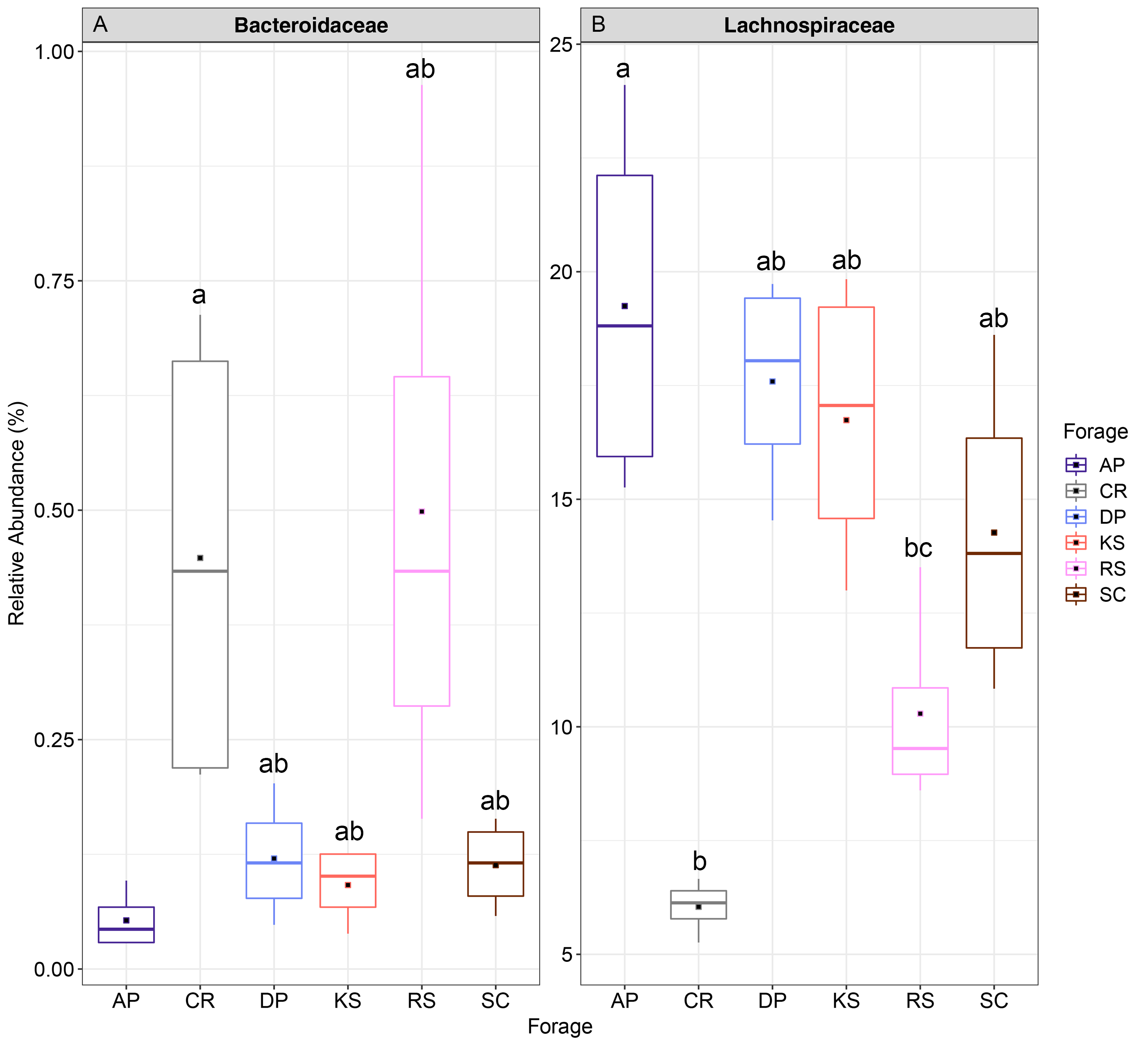

Supplement: Supplemental Information 7 — Differential abundances were statistically tested using ANCOM with a p-value cutoff < 0.05. Means were compared with Duncan post-hoc test only accepting Bonferroni corrected p-values less than 0.05. Boxplots labeled with different letters show statistically significant differences. The solid square shows mean and center line represents median value. AP, camelthorn, CR, common reed; DP, date palm; KS, Kochia; RS, rice straw; and SC, Salicornia. [file peerj-09-10463-s007.png]

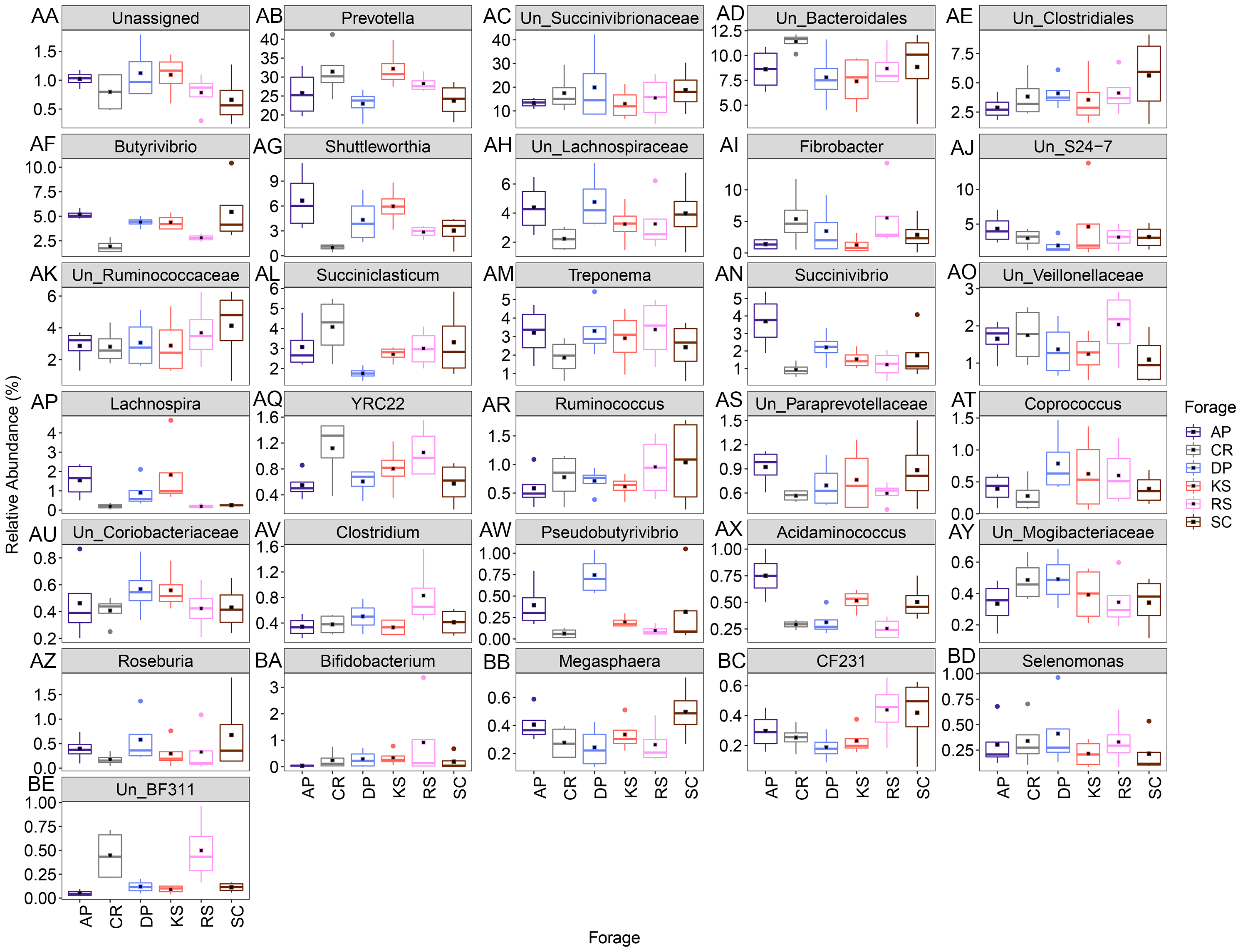

Supplement: Supplemental Information 8 — The solid square shows mean and center line represents median value. AP, camelthorn, CR, common reed; DP, date palm; KS, Kochia; RS, rice straw; and SC, Salicornia. “Un” refers to unclassified. [file peerj-09-10463-s008.png]
