# Supplementary material for: Diversity of microbes colonizing forages of varying lignocellulose properties in the sheep rumen
Source: PeerJ. 2021 Jan 11;9:e10463. doi: 10.7717/peerj.10463 (PMC7808268; doi:10.7717/peerj.10463)
Supplement: Supplemental Information 9 — NDF; neutral detergent fiber, ADF; acid detergent fiber, ADL; acid detergent lignin [file peerj-09-10463-s009.docx]

Table S1:

Chemical composition analysis of six original forages (%DM).

| **ADL** | **ADF** | **NDF** | **ID** | **Name** |
| --- | --- | --- | --- | --- |
| 25.0 | 56.0 | 72.6 | AP | Camelthorn (*Alhagi persarum*) |
| 6.4 | 45.4 | 79.2 | CR | Common reed (*Phragmites australis*) |
| 12.08 | 49.0 | 77.8 | DP | Date palm (*Phoenix dactylifera*) |
| 6.8 | 45.2 | 78.8 | KS | Kochia (*Kochia scoparia*) |
| 3.0 | 47.2 | 77.2 | RS | Rice straw (*Oryza sativa*) |
| 8.0 | 43.2 | 82.0 | SC | Salicornia (*Salicornia persica*) |

NDF; neutral detergent fiber, ADF; acid detergent fiber, ADL; acid detergent lignin
