# Supplementary material for: Diversity of microbes colonizing forages of varying lignocellulose properties in the sheep rumen
Source: PeerJ. 2021 Jan 11;9:e10463. doi: 10.7717/peerj.10463 (PMC7808268; doi:10.7717/peerj.10463)
Supplement: Supplemental Information 10 — DM; dry matter, NDF; neutral detergent fiber, ADF; acid detergent fiber, ADL; acid detergent lignin. Statistically significant differences were determined using one-way ANOVA. Means were compared using Duncan post-hoc test. Different means were denoted using letters at each time point at p < 0.05. AP, camelthorn, CR, common reed; DP, date palm; KS, Kochia; RS, rice straw; and SC, Salicornia. [file peerj-09-10463-s010.docx]

Table S2:
*In sacco* disappearance of chemical composition measured on the six experimental forages.

| Time | Feeds | DM | NDF | ADF | ADL | Cellulose | Hemicellulose |
| --- | --- | --- | --- | --- | --- | --- | --- |
| 24 h after rumen incubation | AP | 30.47±2.40^abc^ | 25.02±1.43^b^ | 23.22±0.41^a^ | 22.73±6.93^a^ | 22.94±1.84^a^ | 31.51±6.30^abcd^ |
|  | CR | 26.20±0.93^c^ | 28.46±0.49^ab^ | 23.67±1.36^a^ | 06.36±4.02^b^ | 25.62±0.34^a^ | 36.37±2.67^abcd^ |
|  | DP | 32.54±1.26^ab^ | 29.18±1.79^ab^ | 23.52±3.70^a^ | 27.66±1.83^a^ | 22.36±4.66^a^ | 39.05±3.50^bc^ |
|  | KS | 33.84±1.92^a^ | 31.00±2.26^a^ | 22.15±2.74^a^ | 26.42±4.35^a^ | 19.80±3.39^a^ | 40.62±2.20^ab^ |
|  | RS | 20.17±0.91^d^ | 16.73±1.38^c^ | 09.85±2.34^b^ | 09.09±3.00^b^ | 10.80±2.00^b^ | 27.12±2.05^d^ |
|  | SC | 28.77±0.82^bc^ | 27.05±1.15^ab^ | 24.82±3.64^a^ | 5.87±3.65^a^ | 26.18±1.45^a^ | 29.73±2.90^cd^ |
| 48 h after rumen incubation | AP | 40.05±4.21^a^ | 32.39±3.21^ab^ | 26.31±3.25^ab^ | 23.58±2.52^cde^ | 28.69±1.27^a^ | 43.92±1.60^ab^ |
|  | CR | 28.85±5.08^cd^ | 30.04±6.97^ab^ | 26.91±7.89^ab^ | 07.89±4.87^b^ | 28.56±9.89^a^ | 40.18±4.01^abc^ |
|  | DP | 41.28±3.13^a^ | 36.29±2.21^a^ | 30.79±0.06^a^ | 37.87±6.95^a^ | 28.76±2.01^a^ | 45.93±5.80^a^ |
|  | KS | 39.40±1.69^ac^ | 32.86±2.01^ab^ | 24.62±5.08^ab^ | 31.08±5.54^ac^ | 24.25±6.85^a^ | 43.66±3.02^ab^ |
|  | RS | 26.28±3.23d | 23.38±2.50^b^ | 17.43±1.94^b^ | 13.64±2.65^eb^ | 18.31±4.58^a^ | 31.26±2.00^cd^ |
|  | SC | 32.94±2.95^acd^ | 29.23±3.09^ab^ | 29.26±2.94^ab^ | 13.78±3.21^bd^ | 28.07±2.31^a^ | 33.62±4.85^bd^ |
| 72 h after rumen incubation | AP | 44.17±4.11^a^ | 33.41±4.07^a^ | 30.22±2.99^a^ | 27.07±4.27 ^bc^ | 32.01±3.41^ab^ | 54.17±4.11^a^ |
|  | CR | 42.30±3.54^ab^ | 33.10±1.09^a^ | 29.89±4.24^a^ | 13.02±6.93 ^b^ | 30.85±1.83^ab^ | 42.30±3.54^bd^ |
|  | DP | 46.71±2.42^a^ | 38.80±1.84^a^ | 34.29±2.97^a^ | 47.16±4.88 ^a^ | 30.59±2.59^ab^ | 46.71±2.42^ab^ |
|  | KS | 45.00±3.98^a^ | 33.73±3.51^a^ | 27.15±4.07^a^ | 33.81±6.93 ^ac^ | 26.36±3.95^ab^ | 49.00±3.98^ab^ |
|  | RS | 31.88±1.84^bc^ | 25.57±9.14^a^ | 21.17±8.31^a^ | 18.18±1.00 ^b^ | 23.07±3.00^b^ | 33.88±1.84^d^ |
|  | SC | 35.15±3.79^ac^ | 31.02±4.50^a^ | 31.28±4.49^a^ | 14.08±3.18 ^b^ | 32.97±5.47^a^ | 35.15±3.79^d^ |
| 96 h after rumen incubation | AP | 43.97±0.93^ab^ | 36.50±3.12^ab^ | 30.48±4.82^a^ | 28.19±7.49^bd^ | 32.36±2.45^a^ | 59.02±3.75^a^ |
|  | CR | 35.34±1.90^ab^ | 35.06±2.13^ab^ | 32.21±2.53^a^ | 14.25±4.13^b^ | 31.16±3.29^a^ | 42.81±1.97^bc^ |
|  | DP | 44.34±2.32^ab^ | 40.48±4.17^a^ | 36.85±3.61^a^ | 48.99±2.75^a^ | 33.35±4.33^a^ | 46.88±5.13^abc^ |
|  | KS | 45.43±1.30^a^ | 39.45±0.86^ab^ | 29.52±0.83^a^ | 35.53±4.94^ad^ | 28.30±0.86^a^ | 54.46±1.53^ab^ |
|  | RS | 32.13±2.44^b^ | 30.50±0.92^b^ | 24.75±2.42^a^ | 18.18±2.65^b^ | 28.61±3.61^a^ | 36.10±2.69^c^ |
|  | SC | 39.88±9.49^ab^ | 34.06±5.68^ab^ | 34.82±0.55^a^ | 16.50±4.60^b^ | 35.20±6.75^a^ | 37.24±9.56^c^ |

DM; dry matter, NDF; neutral detergent fiber, ADF; acid detergent fiber, ADL; acid detergent lignin. Statistically significant differences were determined using one-way ANOVA. Means were compared using Duncan post-hoc test. Different means were denoted using letters at each time point at p < 0.05. AP; camelthorn, CR; common reed, DP; date palm, KS; Kochia, RS; rice straw, and SC; Salicornia.
