# Supplementary material for: Diversity of microbes colonizing forages of varying lignocellulose properties in the sheep rumen
Source: PeerJ. 2021 Jan 11;9:e10463. doi: 10.7717/peerj.10463 (PMC7808268; doi:10.7717/peerj.10463)
Supplement: Supplemental Information 11 — DF; degree of freedom, DM; dry matter, NDF; neutral detergent fiber, ADF; acid detergent fiber, ADL; acid detergent lignin. [file peerj-09-10463-s011.docx]

Table S3:

GLM ANOVA results (F and P values) for the main and interaction effects of forage and incubation time on measured chemical compositions of six experimental forages.

| Hemicellulose | | Cellulose | | ADL | | ADF | | NDF | | DM | | DF | Source |
| --- | --- | --- | --- | --- | --- | --- | --- | --- | --- | --- | --- | --- | --- |
| P | F | P | F | P | F | P | F | P | F | P | F |  |  |
| <0.0001 | 22.89 | 0.0013 | 4.77 | <0.0001 | 50.56 | 0.0015 | 4.65 | 0.0002 | 6.23 | <0.0001 | 33.75 | 5 | Forage |
| <0.0001 | 27.78 | <0.0001 | 16.26 | <0.0001 | 32.67 | <0.0001 | 17.44 | <0.0001 | 19.82 | <0.0001 | 32.52 | 3 | Time |
| 0.0229 | 2.15 | 0.5933 | 0.88 | <0.0001 | 5.46 | 0.2639 | 1.26 | 0.4167 | 1.06 | 0.8363 | 0.63 | 15 | Forage × Time |
| <0.0001 | 10 | <0.0001 | 3.73 | <0.0001 | 18.81 | <0.0001 | 4.11 | <0.0001 | 4.63 | <0.0001 | 11.99 | 23 | Model |

DF; degree of freedom, DM; dry matter, NDF; neutral detergent fiber, ADF; acid detergent fiber, ADL; acid detergent lignin.
