# Supplementary material for: Diversity of microbes colonizing forages of varying lignocellulose properties in the sheep rumen
Source: PeerJ. 2021 Jan 11;9:e10463. doi: 10.7717/peerj.10463 (PMC7808268; doi:10.7717/peerj.10463)
Supplement: Supplemental Information 12 — p = a + b (1 –exp−ct) where, p; is rumen disappearance at time t (h), a; washing losses, soluble or rapidly degradable fraction constant, b; slowly degradable fraction constant, c; degradation rate, t; time of incubation, ED; effective degradability was calculated as a + (b × c)/(c + k) at three ruminal passage rates (k = 0.02, 0.04, and 0.06 h−1), PD; potential degradability calculated as a+b. Statistically significant differences were determined using one-way ANOVA. Means within row, were compared using Duncan post-hoc test. Different means were denoted using letters for each forage at Bonferroni corrected p < 0.05. AP, camelthorn, CR, common reed, DP, date palm; KS, Kochia; RS, rice straw; and SC, Salicornia. DM; dry matter, NDF; neutral detergent fiber, ADF; acid detergent fiber, SEM; standard error of the mean. [file peerj-09-10463-s012.docx]

Table S4:

Degradation kinetics for chemical composition of the six experimental forages evaluated by the exponential model (Orskov and McDonald, 1979^&^).

| SEM | Forages | | | | | | Degradation kinetics | Forage |
| --- | --- | --- | --- | --- | --- | --- | --- | --- |
|  | **SC** | **RS** | **KS** | **DP** | **CR** | **AP** |  |  |
|  |  |  |  |  |  |  |  |  |
| 2.616 | 23.54^b^ | 2.00^d^ | 29.08^a^ | 2.78^d^ | 15.10^c^ | 3.61^d^ | a | **DM** |
| 2.202 | 33.61^ab^ | 29.34^ab^ | 32.03^ab^ | 43.72^a^ | 22.74^b^ | 41.54ab | b |  |
| 3.219 | 57.15^ab^ | 31.34^b^ | 61.11^a^ | 46.50^ab^ | 37.85^ab^ | 45.15ab | PD |  |
| 0.007 | 0.007^b^ | 0.078^d^ | 0.007^b^ | 0.054^ad^ | 0.010^b^ | 0.043a | c |  |
| 2.031 | 32.20^ab^ | 16.00^b^ | 37.50^a^ | 32.70^ab^ | 27.90^ab^ | 32.00^ab^ | ED2 |  |
| 2.106 | 28.50^b^ | 9.10^a^ | 33.90^b^ | 25.90^b^ | 25.80^b^ | 25.20^b^ | ED4 |  |
| 2.236 | 27.00^b^ | 5.40^a^ | 32.50^b^ | 21.50^b^ | 25.00^b^ | 21.00^b^ | ED6 |  |
|  |  |  |  |  |  |  |  | **NDF** |
| 1.955 | 24.30^a^ | 7.62^b^ | 24.17^ad^ | 12.92^bd^ | 25.86^a^ | 10.22^b^ | a |  |
| 2.392 | 49.60^a^ | 25.19^b^ | 33.06^ab^ | 28.34^b^ | 34.36^ab^ | 26.65^b^ | b |  |
| 4.095 | 73.89^a^ | 32.80^b^ | 57.23^ab^ | 41.26^b^ | 60.22^ab^ | 36.87^b^ | PD |  |
| 0.003 | 0.002^c^ | 0.019^b^ | 0.006^c^ | 0.035^a^ | 0.003^c^ | 0.0342^a^ | c |  |
| 1.515 | 29.40^a^ | 19.90^a^ | 32.30^a^ | 31.10^a^ | 30.60^a^ | 27.00^a^ | ED2 |  |
| 1.482 | 27.00^a^ | 15.80^a^ | 28.80^a^ | 26.30^a^ | 28.40^a^ | 22.50^a^ | ED4 |  |
| 1.518 | 26.10^a^ | 13.70^a^ | 27.40^a^ | 23.50^a^ | 27.60^a^ | 19.90^a^ | ED6 |  |
|  |  |  |  |  |  |  |  | **ADF** |
| 1.671 | 20.79^d^ | 2.38^c^ | 17.07^ad^ | 10.88^abc^ | 20.19^ad^ | 16.86^ad^ | a |  |
| 3.596 | 32.11^acf^ | 49.46^abc^ | 22.19^f^ | 28.59^df^ | 53.76^ac^ | 16.50^f^ | b |  |
| 3.807 | 52.89^ab^ | 51.85^ab^ | 39.26^b^ | 39.47^b^ | 73.95^a^ | 33.36^b^ | PD |  |
| 0.012 | 0.006^d^ | 0.007^d^ | 0.005^d^ | 0.024^a^ | 0.003^d^ | 0.019^a^ | c |  |
| 1.416 | 28.10^a^ | 15.30^a^ | 23.70^a^ | 26.60^a^ | 26.80^a^ | 25.00^a^ | ED2 |  |
| 1.470 | 24.90^a^ | 9.80^b^ | 21.00^ab^ | 21.70^ab^ | 23.70^a^ | 22.30^ab^ | ED4 |  |
| 1.503 | 23.60^a^ | 7.60^b^ | 19.80^a^ | 19.20^ab^ | 22.60^a^ | 20.90^a^ | ED6 |  |

*^&^*p = a + b (1 – exp^-ct^) where, p; is rumen disappearance at time t (h), a; washing losses, soluble or rapidly degradable fraction constant, b; slowly degradable fraction constant, c; degradation rate, t; time of incubation, ED; effective degradability was calculated as a + (b × c)/(c + k) at three ruminal passage rates (k= 0.02, 0.04, and 0.06 h^-1^), PD; potential degradability calculated as a+b. Statistically significant differences were determined using one-way ANOVA. Means within row, were compared using Duncan post-hoc test. Different means were denoted using letters for each forage at Bonferroni corrected p < 0.05. AP; camelthorn, CR; common reed, DP; date palm, KS; Kochia, RS; rice straw, and SC; Salicornia. DM; dry matter, NDF; neutral detergent fiber, ADF; acid detergent fiber, SEM; standard error of the mean.
