# Supplementary material for: Diversity of microbes colonizing forages of varying lignocellulose properties in the sheep rumen
Source: PeerJ. 2021 Jan 11;9:e10463. doi: 10.7717/peerj.10463 (PMC7808268; doi:10.7717/peerj.10463)
Supplement: Supplemental Information 13 — Linear i ; linearly increased, Linear d ; linearly Kochia; RS, rice straw; and SC, Salicornia. [file peerj-09-10463-s013.docx]

Table S5:

Testing for distributional trend of ruminal microbiome (genus level) adhered to the six lignocellulosic forages during their rumen incubation (over 96 h with 24 h intervals) using orthogonal contrast analysis.

| **Forage** | **Genus** | **Trend** | **F value** | **Pr > F** |
| --- | --- | --- | --- | --- |
| AP | Prevotella | Linear*_d_* | 7.88 | 0.0262 |
|  | Shuttleworthia | Quadratic | 6.68 | 0.0362 |
|  | Selenomonas | Linear*_d_* | 14.64 | 0.0065 |
| CR | Selenomonas | Quadratic | 6.73 | 0.0319 |
| DP | Un_Succinivibrionaceae | Cubic | 5.58 | 0.0458 |
|  | Fibrobacter | Linear*_i_* | 11.89 | 0.0087 |
|  | Coprococcus | Cubic | 6.98 | 0.0296 |
|  | Selenomonas | Quadratic | 14.85 | 0.0049 |
| KS | Fibrobacter | Linear*_i_* | 6.92 | 0.0302 |
|  | Un_S24-7 | Quadratic | 4.86 | 0.0334 |
|  | Un_Veillonellaceae | Quadratic | 11.71 | 0.0091 |
|  | Lachnospira | Cubic | 8.49 | 0.0195 |
|  | Un_Paraprevotellaceae | Cubic | 7.37 | 0.0264 |
| RS | Fibrobacter | Linear*_i_* | 8.37 | 0.0201 |
|  | Un_S24-7 | Quadratic | 4.82 | 0.0500 |
|  | Un_Veillonellaceae | Quadratic | 11.58 | 0.0093 |
|  | Ruminococcus | Cubic | 10.97 | 0.0107 |
|  | Coprococcus | Cubic | 7.04 | 0.0291 |
|  | Roseburia | Linear*_d_* | 5.13 | 0.0492 |
| SC | Butyrivibrio | Linear*_d_* | 14.89 | 0.0062 |
|  | Fibrobacter | Linear*_i_* | 6.32 | 0.0402 |
|  | Un_S24-7 | Linear*_i_* | 6.52 | 0.0379 |
|  | Treponema | Cubic | 13.03 | 0.0086 |
|  | Un_Veillonellaceae | Quadratic | 34.82 | 0.0006 |
|  | Un_Paraprevotellaceae | Linear*_i_* | 13.75 | 0.0076 |
|  | Roseburia | Quadratic | 11.53 | 0.0115 |
|  | Bifidobacterium | Linear*_d_* | 5.69 | 0.0485 |
|  | Selenomonas | Linear*_d_* | 9.92 | 0.0162 |

Linear*_i_* ; linearly increased, Linear*_d_* ; linearly decreased, AP; camelthorn, CR; common reed, DP; date palm, KS; Kochia, RS; rice straw, and SC; Salicornia.
